# The autonomic brain: Multi-dimensional generative hierarchical modelling of the autonomic connectome

**DOI:** 10.1016/j.cortex.2021.06.012

**Published:** 2021-10

**Authors:** James K. Ruffle, Harpreet Hyare, Matthew A. Howard, Adam D. Farmer, A. Vania Apkarian, Steven C.R. Williams, Qasim Aziz, Parashkev Nachev

**Affiliations:** a Queen Square Institute of Neurology, University College London, UK; b Department of Radiology, University College London Hospital NHS Foundation Trust, London, UK; c Centre for Neuroscience and Trauma, Blizard Institute, Wingate Institute of Neurogastroenterology, Barts and the London School of Medicine & Dentistry, Queen Mary University of London, London, UK; d Department of Neuroimaging, King's College London, Institute of Psychiatry, Psychology & Neuroscience, London, UK; e Department of Physiology, Northwestern University, Feinberg School of Medicine, Chicago, IL, USA; f Department of Gastroenterology, University Hospitals Midlands NHS Trust, Stoke on Trent, Staffordshire, UK; g Institute of Applied Clinical Sciences, University of Keele, Keele, UK

**Keywords:** Autonomic nervous system, Parasympathetic nervous system, Sympathetic nervous system, Brain imaging, Brain structure, Brain function, Heart rate variability, Generative networks

## Abstract

The autonomic nervous system governs the body's multifaceted internal adaptation to diverse changes in the external environment, a role more complex than is accessible to the methods—and data scales—hitherto used to illuminate its operation. Here we apply generative graphical modelling to large-scale multimodal neuroimaging data encompassing normal and abnormal states to derive a comprehensive hierarchical representation of the autonomic brain. We demonstrate that whereas conventional structural and functional maps identify regions jointly modulated by parasympathetic and sympathetic systems, only graphical analysis discriminates between them, revealing the cardinal roles of the autonomic system to be mediated by high-level distributed interactions. We provide a novel representation of the autonomic system—a multidimensional, generative network—that renders its richness tractable within future models of its function in health and disease.

## 1 Introduction

The autonomic nervous system is a bi-directional brain-body interface, maintaining homeostasis by adapting the internal environment in response to the demands of the external. Comprised of two principal divisions—the sympathetic and the parasympathetic—it regulates a wide array of bodily processes, ranging from breathing, circulation, metabolism, inflammation, to pain and sensation (Bonaz, Picq, Sinniger, Mayol, & Clarencon, 2013; Botha et al., 2015; Frokjaer et al., 2016; Matteoli et al., 2014). Autonomic function is perturbed naturally across a vast range of disorders spanning numerous body systems, including the neurological, cardiorespiratory, gastrointestinal and rheumatological, as well as iatrogenically from drugs and interventions (Benarroch, 1993; Mathias, 2003). So multifaceted a physiological role, with so many points of pathological vulnerability, requires the closest, most detailed evaluation. Yet the fundamental organisation of the autonomic nervous system has hitherto been studied only with methods—and on data scales—that do not permit a finely specified, high-dimensional characterisation.

In recent decades, multiple brain imaging studies have investigated the neural correlates of the autonomic nervous system. The ‘central autonomic network’ (Benarroch, 1993; Critchley & Harrison, 2013) that has emerged, however, is essentially drawn across small (rarely over 30 subjects), typically young, homogeneous cohorts, comfortably within the bounds of healthy normality, studied along a single dimension (see table 1 of ref (Beissner, Meissner, Bar, & Napadow, 2013)). For example, one characteristic (such as regional neural activity) may be investigated with one imaging modality (such as functional magnetic resonance imaging (fMRI)), within a small healthy cohort, often of the same sex and/or within a single decade of life (Goswami, Frances, & Shoemaker, 2011; Nugent, Bain, Thayer, Sollers, & Drevets, 2011). Though valuable in disclosing generalities of neural organisation, this approach has limited power to capture structural and functional aspects that are constitutionally heterogeneous, especially as they cross into the pathological realm. Representing biological heterogeneity in greater detail, and with greater precision, permits pathological changes to be more sensitively detected, for they then become more easily distinguished from normal variation. A single dimensional approach to investigating the autonomic nervous system is furthermore blind to the *interactions* between distinct characteristics such as white matter architecture and grey matter concentrations, leaving in the dark aspects of physiology and pathology that primarily manifest in this way.

Moreover, the central autonomic network, while called a ‘network’, is largely based upon studies that preceded the development of modelling techniques for studying the brain formally as a *graph* (such as with functional connectivity and network statistics (Bullmore & Sporns, 2009)). Even within single dimensions such as white matter architecture, it may be that connectivity between elements—an approach only a graphical analysis could reveal (Bullmore & Sporns, 2009; Sporns, 2013)—is most informative, both in explaining normal function and characterising its pathological deviation.

These arguments compel a new, integrative approach to defining the human autonomic nervous system that surveys the autonomic brain at high resolution, along multiple interacting dimensions, within a highly expressive generative graphical model applied to a large and diverse population encompassing the spectrum of autonomic health and the deviation from it.

Here we prototype such an approach with hierarchical stochastic block modelling of the largest and most detailed set of autonomic and neuroimaging data ever examined. We seek to address five key aspects of the neural substrates of autonomic function responsive to resting heart rate variability (HRV).

First, we determine the differences between conventional unimodal and graphical multimodal representations of autonomic function, identifying the features only graphical analysis sensitive to complex interactions between areas can illuminate. If the two approaches yield identical maps, graphical analysis is superfluous, if they are different, the approach that produces the most extensive, robustly distinctive maps ought to be preferred, for a broader swathe of relevant substrates is thereby implicated.

Second, we identify characteristic macroscopic neural “communities” of multiple areas exhibiting similar patterns of inter-relatedness. This casts light on the fundamental organisation of autonomic function, generating hypotheses explicitly testable in subsequent, focused studies.

Third, we derive a hierarchical parcellation of the brain into regions-of-interest optimally tuned to detecting differences between areas implicated in autonomic function. Such domain-specific parcellation can facilitate the design and execution of group comparisons of morphological differences—innate or acquired.

Fourth, we provide a principled mechanism for integrating multimodal information in quantifying the impact of regional dysfunction—such as focal brain injury may cause—for the purposes of individual clinical outcome prediction or individual therapeutic effect estimation, again for use in downstream predictive or prescriptive studies.

Fifth, we demonstrate the application of generative graphical modelling to large-scale multimodal data, enabling others to extend it, both within the autonomic domain and outside it.

## 2 Materials and methods

### 2.1 Study population

**Fig. 1:**
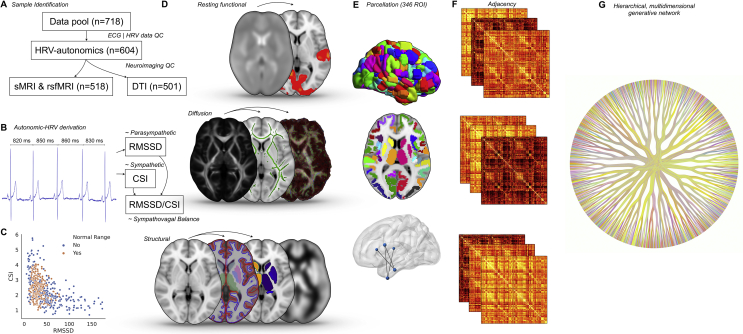
Processing pipeline. A) The original ECG-containing data pool consisted of 718 individuals. After signals processing and quality control of heart rate variability (HRV) data, 604 individuals with viable autonomic data remained. After pre-processing and quality control of neuroimaging data, there were 518 individuals with viable functional and structural imaging, and 501 of those with viable diffusion imaging. B) Signal processing of ECG data was employed to elucidate autonomic tone by HRV, specifically the markers of cardiac sympathetic index (CSI) [∼sympathetic], root mean square of successive differences (RMSSD) [∼parasympathetic] and the ratio between the two (RMSSD/CSI) as an approximation of sympathovagal balance. C) Scatter plot of joint distribution between RMSSD (∼Parasympathetic) and CSI (∼Sympathetic), where participants whose autonomic tone were within normal range and outside of it are colour-coded in orange and blue, respectively. D) Neuroimaging data utilized were as follows: i) functional magnetic resonance imaging - resting activity and connectivity; ii) diffusion tensor imaging – tract based spatial statistics and probabilistic tractography; and iii) structural imaging – analysis of cortical thickness and volume, morphometry of the subcortex and gray matter voxel-based morphometry. E) Connectivity matrices of functional activity, white matter tractography and gray matter morphometry were generated using a validated parcellation scheme of the cortex (Gordon et al., 2016), supplemented with the subcortex. These were statistically evaluated with network-based statistics to generate weighted adjacency matrices of an undirected graph (F), which would then be used in multi-dimensional generative networks to identify connectomes implicated in resting autonomic tone. G) Example of this approach with a single human brain.

Patient data were obtained from the Cambridge Centre for Ageing and Neuroscience (Cam-CAN) repository (Shafto et al., 2014; Taylor et al., 2017), an open dataset of 3000 participants aimed to evaluate healthy cognitive ageing. We report how we determined our sample size, all data exclusions, all inclusion/exclusion criteria, whether inclusion/exclusion criteria were established prior to data analysis, all manipulations, and all measures in the study. The conception of this study did not anticipate our analysis and is not informed by our aims. Our inclusion criteria were those with both MRI brain imaging and electrocardiographic (ECG) tracing, in order to allow evaluation of autonomic activity by HRV and to ascertain its corresponding neural correlate. We excluded all participants with major pre-existing medical diagnoses, including cardiovascular, respiratory, neurological or psychiatric conditions, in addition to those taking medication. This design sought to capture the spectrum of autonomic normality and its distributional tails to abnormality, with minimal confounding from substantial disruption of other bodily systems. Further exclusion criteria were individuals with ECG tracings under 5 min in total length, the rationale of which was to ensure that derivation of HRV-based autonomic measures were in accordance with a minimum 5-minute epoch, aligning to international guidelines (European, Society, of, & Cardiology, 1996). Participants with a previously undiagnosed dysrhythmia noted on ECG trace were also excluded. We excluded any participants who did not have a full set of cardiac observations, including blood pressure, as we decided *a priori* to model for blood pressure as an additional nuisance covariate. For brain imaging, we excluded any studies with demonstrable artefact, anatomical abnormality, movement exceeding 1 mm in either x, y or z translation or rotational movement greater than 1° around any axis, during the entire fMRI sequence. This culminated in five hundred and eighteen samples with suitable functional and structural brain imaging data available (261 males and 257 females, cohort mean ± SEM age 53.25 ± .79, range 18–88). For diffusion tensor imaging (DTI), a further seventeen participants were excluded due to sub-optimal image quality (251 males and 250 females, mean age 53.44 ± .81, range 18–88). A processing pipeline for the study methods is visualised as Fig. 1.

### 2.2 Ethical approval

The Cam-CAN project was approved by local ethics committee, Cambridgeshire Research Ethics Committee (reference: 10/H0308/50), and our study submission to the Cam-CAN group was approved by their departmental committee in late 2017. All participants for the Cam-CAN study gave written consent (Shafto et al., 2014; Taylor et al., 2017).

### 2.3 Autonomic measure pre-processing

We used HRV – a quantifiable aspect of autonomic function commonly examined in neuroscience and brain imaging (Beissner et al., 2013) – as the source of a set of summary indices of inter-individual variation in autonomic activity. A comprehensive set of measures of autonomic function would be infeasibly large for a dataset of this size, and our concern here is in any event with the correlates of synoptic functional “signatures” of sympathetic and parasympathetic activity that can be specified compactly. All autonomic analysis were undertaken as per the international guidelines provided by the European Society of Cardiology and the North American Society of Pacing Electrophysiology (European et al., 1996). Cardiac data were acquired homogenously in all subjects, from the physiological signals channel of magnetoencephalography (MEG) using a pair of bipolar electrodes to record ECG signal, as described in (Shafto et al., 2014), acquired while participants sat within a 306-channel Vectorview system (Elekta Neuromag, Helsinki, Finland), consisting of 102 magnetometers and 204 orthogonal planar gradiometers (see (Shafto et al., 2014; Taylor et al., 2017)). Data was sampled at 1000 Hz with a band-pass filter of .03–330 Hz. Raw ECG waveform data from MEG channels were extracted using Statistical Parametric Mapping 8 (University College London). Using MATLAB (2018a), an automated pre-processing pipeline was developed to ensure homogenous signal pre-processing of cardiac-autonomic data, and to avoid probable human error. Notably, this included the determination of a QRS complex as genuine or artefactual, which can otherwise be a major source of human error in analysis. Individual pipeline constituents were employed using modifications of the freely available code in HRVTool (https://github.com/MarcusVollmer/HRV) (Vollmer, 2015, 2020). The first 20 s of the ECG recording was always discarded to allow for signal stabilization in all samples. The remaining duration of ECG monitoring periods varied across participants (median 9.38 min, range 5 - 18.35 min), and thus were subsampled to 5 min to ensure analytical homogeneity temporally (European et al., 1996). QRS peak detection were employed and inter-beat intervals (RR interval) calculated, in milliseconds. Artefact removal was performed using the automated HRVTool filter. Pre-processed traces were outputted to pictorial display for the investigator to then review manually, for quality control purposes. HRV measures used were the following: 1) root mean square of successive differences (RMSSD) as a surrogate to parasympathetic tone (European et al., 1996); 2) cardiac sympathetic index (CSI) as a surrogate of sympathetic tone (Allen, 2002; Allen, Chambers, & Towers, 2007; Toichi, Sugiura, Murai, & Sengoku, 1997); and 3) RMSSD/CSI, the ratio between the two, as a surrogate to sympathovagal balance (supplementary material) (joint distribution available in Fig. 1). The justification for the use of these specific measures as surrogate markers to autonomic tone were on the review of selecting HRV metrics least affected by other possible physiological confounds, as per (Laborde, Mosley, & Thayer, 2017). The computational derivation and validation of these measures is described in detail elsewhere (Allen, 2002; Allen et al., 2007; European et al., 1996; Shaffer & Ginsberg, 2017; Vollmer, 2015). Any values that exceeded ± two standard deviations of the cohort were automatically flagged, triggering a secondary ECG tracing review of these participants, to ensure no arrhythmias were demonstrable that might otherwise confound results. We noted that, whilst the utility of RMSSD is well validated as a marker of parasympathetic tone across decades of autonomic neuroscience research, a marker for sympathetic tone from HRV remains less well characterised, with different research groups using different markers. Based upon literature review of HRV markers for sympathetic tone (Allen, 2002; Allen et al., 2007; Goldstein, Bentho, Park, & Sharabi, 2011), we decided to use CSI as the most plausible HRV-derived approximation (Toichi et al., 1997). While this marker may be further from the ‘ground truth’ than invasive measurements of sympathetic nerve activity (Macefield & Henderson, 2019), it is the only logistically feasible option at this data scale.

ECG and HRV measures were deliberately acquired in a session separate from brain imaging. This is because our focus here is the tonic, resting-state characteristics of autonomic balance that the noise and claustrophobia of the MR imaging environment would render unnatural and unrepresentative. A previous study has shown that such ‘snapshot’ quantification of resting autonomic measures is representative of typical autonomic tone, and reproducible over 1 year later (Farmer et al., 2014). Over the median duration between HRV acquisition and MRI scanning of 42 days, it would be reasonable to expect no material change in grey or white matter organisation perceptible at the group level. Resting state functional data also shows good stability over time (Choe et al., 2015; Finn et al., 2015).

### 2.4 Acquisition of MRI data

All MRI data reported here were acquired by the CamCAN group (Shafto et al., 2014; Taylor et al., 2017), according to a protocol designed without our input. All MRI data were acquired homogenously in a one-hour session conducted at the MRC-CBSU using a 3 Tesla Siemens TIM Trio System, with 32-channel head coil. Sequences acquired that were used in our analysis were the following: 1) T1-Weighted Structural Image: a high-resolution 3D image were acquired using the Magnetization Prepared Rapid Gradient Echo (MPRAGE) sequence with the following parameters: Repetition Time (TR) 2250 msec; Echo Time (TE) 2.99 msec; Inversion Time (TI) 900 msec; flip angle 9°; Field of View (FOV) 256 mm × 240 mm x 192 mm; voxel size 1 mm isotropic; GRAPPA acceleration factor 2; acquisition time of 4 min and 32 s 2) Diffusion-Weighted Images (DWI): DWI were acquired with a twice-refocused spin-echo sequence, with 30 diffusion gradient directions for each of two b-values: 1000 and 2000 sec/mm^2^, plus three images acquired with a b-value of 0. These parameters are optimised for estimation of the diffusion kurtosis tensor and associated scalar metrics, as well as the traditional diffusion tensor. Other parameters as follows: TR 9100 msec; TE 104 msec; voxel size 2 mm isotropic; FOV 192 mm × 192 mm, 66 axial slices, number of averages 1; acquisition time of 10 min and 2 s 3) Resting state functional MRI (rsfMRI): T2∗-weighted images were acquired during which participants rest with their eyes shut using a Gradient-Echo Echo-Planar Imaging (EPI) sequence. A total of 261 volumes were acquired, each containing 32 axial slices (acquired in descending order), slice thickness 3.7 mm with interslice gap of 20%, providing whole brain coverage including cerebellum; TR 1970 msec; TE 30 msec; flip angle 78°; FOC 192 mm × 192 mm; voxel size 3 mm × 3 mm x 4.44 mm, acquisition time of 8 min and 40 s. Full documentation of MR protocols are provided by the CamCAN group (Shafto et al., 2014; Taylor et al., 2017).

### 2.5 Pre-processing and statistical analysis of neuroimaging data

Before any pre-processing proper, all sequences were carefully reviewed manually for signal and image artefact that may have otherwise confounded findings. Images excluded are documented above in *Study Population*. All scans were re-reviewed at every pre-processing stage to ensure no fault which may confound findings.

#### 2.5.1 Structural MRI

Structural imaging was pre-processed for three specific domains: i) *Cortex-specific measures* were pre-processed using FreeSurfer (http://surfer.nmr.mgh.harvard.edu/) (Dale, Fischl, & Sereno, 1999), specifically for analysis of cortical thickness and cortical volumes; ii) *Subcortex-specific measures* were pre-processed and analysed using the FMRIB Software Library (FSL) FIRST analysis package (Patenaude, Smith, Kennedy, & Jenkinson, 2011), for analysis of subcortical morphology and volumetry and iii) *Whole-brain voxel brain morphometry (VBM)* were pre-processed using the CAT-12 (http://www.neuro.uni-jena.de/cat/) adaptation of VBM within SPM12(Ashburner & Friston, 2005) for generation of gray-matter morphometric networks (Supplementary Material).

#### 2.5.2 Resting-state functional MRI

*Pre-processing of fMRI:* was undertaken using the FMRI Expert Analysis Tool (FEAT), version 6.00, within FSL (Smith et al., 2004). The first 4 volumes of each 4-dimensional fMRI time-series were always discarded to allow for signal stabilisation. The following pre-statistical processing steps were applied: Motion Correction with FMRIB Linear Image Registration Tool (MCFLIRT) (7 degrees of freedom); slice-timing correction using Fourier-space time-series phase-shifting; brain extraction (BET); spatial smoothing using a Gaussian kernel of full-width-half-maximum (FWHM) 5 mm; grand-mean intensity normalisation of the entire 4-dimensional dataset by a single multiplicative factor; high pass temporal filtering (Gaussian-weighted least-squares straight line fitting, with sigma = 50.0s); registration to high resolution structural and standard space images using the FMRIB Linear Image Registration Tool (FLIRT) (Jenkinson, Bannister, Brady, & Smith, 2002).

#### 2.5.3 Diffusion weighted imaging

*Pre-processing of DWI:* were performed using the FMRIB diffusion toolbox (FDT). Using TBSS(Smith et al., 2006), Fractional Anisotropy (FA) images were created by fitting a tensor model to raw diffusion data using FDT, in addition to skull-stripping. All subject's FA data were aligned into common space using nonlinear registration (FNIRT), which uses a b-spline representation of the registration warp field. Subsequently, the mean FA image was created and thinned to create a mean FA skeleton, which represents the centres of all tracts common to the group. Each subject's aligned FA data was projected onto this skeleton and the resulting data fed into voxelwise cross-subject statistics. Results were cross-referenced with white matter/tract atlases (Tang, Sun, Toga, Ringman, & Shi, 2018) as appropriate. *Tractography:* were performed using GPU implementations of bedpostx and probtrackx2 (Hernandez-Fernandez et al., 2019).

#### 2.5.4 Whole brain parcellations

For network connectivity analyses, parcellation of brain data were undertaken using the (Gordon et al., 2016) schema of 333 cortical regions, further supplemented by the inclusion of 13 subcortical regions to form a parcellation scheme of 346 unique regions (parcel list provided as Supplementary Data, the original surface parcellation defined is available here (Gordon et al., 2016)). Functional connectivity adjacency matrices were generated by extraction of time-series blood oxygen level dependent (BOLD)-signal for each region with subsequent pairwise correlation coefficient ascertained with r to z transformation, white matter matrices by probabilistic tractography of streamlines (normalised by waypoint), and gray matter-morphometric matrices using GrayNet (https://github.com/raamana/graynet) (Raamana & Strother, 2018) where, using the Manhattan method, histogram distance between cortical thickness and subcortical volumes of the parcellation were ascertained. For each subject, and for each imaging modality, these approaches produced adjacency matrices of 59,685 edges.

#### 2.5.5 Statistical analysis

General linear modelling (GLM) was used to statistically evaluate the effects of the autonomic nervous system on brain gray matter structure (cortical thickness, cortical volumes, subcortical morphometry, subcortical volumes), white matter (tract based spatial statistics) and resting functional activity. These were performed with permutation tested non-parametric inference, using FSL-RANDOMISE. Tests were linear contrasts for effect of i) RMSSD, ii) CSI and iii) RMSSD/CSI, all of which also included nuisance covariates of age, gender, mean arterial pressure, with variable de-meaning as per (Mumford, Poline, & Poldrack, 2015). When a given autonomic variable was tested, all other autonomic measures were also included as nuisance regressors. For structure-based analyses, total intracranial volume was also included as an additional nuisance regressor. For example, a general linear model of subcortical morphometry testing the effect of RMSSD would feature the following nuisance covariates: CSI, RMSSD/CSI, age, gender, mean arterial pressure and total intracranial volume. Post-hoc correction was employed by virtue of threshold-free-cluster-enhancement (TFCE) and false discovery rate (FDR) where appropriate, to which the significance value for all findings reported are to a significance threshold of *corrected*-*p* < .05. Neuroimaging data were visualised with BrainNet (Xia, Wang, & He, 2013).

#### 2.5.6 Network analysis of multi-modal brain networks

Resting functional, white matter tractography and gray-matter morphometric networks, contingent on resting autonomic tone were analysed by the network based statistics (NBS) connectome toolbox (v1.2 (Zalesky, Cocchi, Fornito, Murray, & Bullmore, 2012; Zalesky, Fornito, & Bullmore, 2010)). The NBS is a non-parametric statistical method which corrects for multiple comparisons and controls for family-wise error and is further discussed in the supplementary material. For all models using the NBS, input data were the graph matter morphometric networks, waytotal normalised tractography matrices and functional connectivity matrices of each participant, with testing of RMSSD, CSI and RMSSD/CSI and inclusion of all nuisance covariates as stated above.

#### 2.5.7 Multi-dimensional, generative, hierarchical, brain networks

Significant and *t*-statistic weighted connectivity matrices from aforementioned network-based statistics were extracted and incorporated into Bayesian weighted, non-parametric, hierarchical, generative stochastic block models (Peixoto, 2018). Generative graphical modelling of brain networks was undertaken using graph-tool (https://graph-tool.skewed.de). Our aim was to identify a high-dimensional connectome built by the culmination of white, gray and functional connectivity matrices representative of and weighted by relationship to resting autonomic tone. A graphical representation of this generative and iterative process is illustrated as Supplementary Movie 1. We compared community structure by normalized mutual information. Network centrality measures were computed by these weighted matrices, including eigenvector centrality, a measure of overall influence of a node with respect to the overall network (Newman, 2008). Further network-based metrics, including small world propensity, were quantified using the brain connectivity toolbox (https://sites.google.com/site/bctnet/) (Bullmore & Sporns, 2009; Rubinov & Sporns, 2010). A given node may belong to more than one network—the communities are not exclusive—what defines them is not the nodal membership but the nature of the connectivity.

Supplementary video related to this article can be found at https://doi.org/10.1016/j.cortex.2021.06.012

The following is/are the supplementary data related to this article:

Multimedia component 12 Multimedia component 12

Multimedia component 13 Multimedia component 13

#### 2.5.8 Hierarchical, graph-delineated, parcellations

Lastly, we utilized the stochastic block model to generate a hierarchical parcellation scheme representative of autonomic tone. The fundamental parcellation was first drawn from the stochastic block model, and further strengthened by sampling from the posterior distribution with iterative Markov chain Monte Carlo equilibration (MCMC) to evidential equilibration based upon model entropy, the state of negative log-likelihood of the microcanonical stochastic block model (Peixoto, 2014), using Metropolis-Hasting acceptance-rejection sampling (Hastings, 1970). This value is also referred to the description length of the data, corresponding to the amount of information required to describe it in nats. We did not specify a finite number of draws, rather we specified a wait step of 1000 iterations for a record-breaking event, to ensure that equilibration was driven by changes in the entropy criterion, instead of driven by a finite number of iterations. Our approach does not utilize burn-in. Rather, we used the generative stochastic block model to initialize the Markov chain from ground state, from which our posterior sampling method ensued as aforementioned. Both nested and non-nested models were constructed, with entropy after MCMC equilibration used to inform of the most plausible fit. We used automated labelling of revealed community structures by the predominant functional community in accordance to well published networks (Gordon et al., 2016).

Having generated a hierarchical graphical representation of autonomic tone guided by functional connectivity, tractography and gray-matter morphometric networks, we used meta-analytic functional maps derived from natural language processing of published imaging studies to assign candidate functional labels to each level of organisation. This was achieved with NeuroQuery image search matching (Dockes et al., 2020). Passing each identified region of interest (ROI) to NeuroQuery repository of 13,459 studies encompassing 5,144 activation pattern terms, we retrieved the closest matching 10 topic terms, at each hierarchical level. We filtered terms to exclude those that were either anatomical, experimental, vague or disease descriptors, utilising the Terminologia Anatomica dictionary to automatically filter out anatomical terms (FIPAT, 2019). This yielded a list of terms for each ROI, each term with a matching score, from which we generated a corpus for each hierarchical level, with detailed information on the term frequency at all hierarchical levels. We de-meaned the matching score across clusters to filter out for commonalities and used word clouds to generate visualisations illustrating the proportionate frequency of terms.

### 2.6 Data and code availability

No part of the study procedures or analyses was preregistered prior to the research being conducted. All brain imaging data for this study is downloadable via the Cam-CAN data repository at https://www.cam-can.org. All software used is all freely available via original referenced sources in the methods. Code excerpts are provided at https://osf.io/s3nej/.

## 3 Results

### 3.1 The imaging signature of the autonomic network

We sought to reveal the interplay between brain structure and function in the operation of the autonomic nervous system as reflected in heart rate variability, within a set of highly expressive statistical models. We examined three key domains, crucially including their complex interactions: i) gray matter structure; ii) white matter structure and iii) resting function, both with conventional univariate-voxel and network-based analysis. A complex array of distinctive characteristics was revealed within each domain, but the most striking differentiation between sympathetic and parasympathetic systems was observed at the network level, further amplified when these imaging domains were jointly modelled, yielding a comprehensive multimodal hierarchical model.

### 3.2 Autonomic measures

Resting mean autonomic measures were acquired by validated heart rate variability metrics in a group of 518 participants (261 males and 257 females, cohort mean ± SEM age 53.25 ± .79, range 18–88). Autonomic measures were in keeping with the established population distribution of normality (n = 277) and the distributional tails of abnormality (n = 241) (Allen, 2002; Shaffer & Ginsberg, 2017; Toichi et al., 1997; Umetani, Singer, McCraty, & Atkinson, 1998): root mean square of successive differences (RMSSD) (broadly parasympathetic) was 44.35 ± 1.43 and cardiac sympathetic index (CSI) was 2.33 ± .04, demonstrating that our sample was reasonably representative. These measures had only a modest anticorrelated relationship (cubic polynomial fit, R^2^ = .30), leaving substantial mutually unexplained variance (Fig. 1). Mean resting heart rate was 63 ± .42. Mean height was 170.50 cm ± .54, whilst mean weight were 74.89 kg ± .67. Blood pressure readings were as follows: i) systolic 120.00 mmHg ± .79; ii) diastolic 73.29 mmHg ± .49 and iii) mean arterial 89.03 mmHg ± .51.

### 3.3 Gray matter structure

**Fig. 2:**
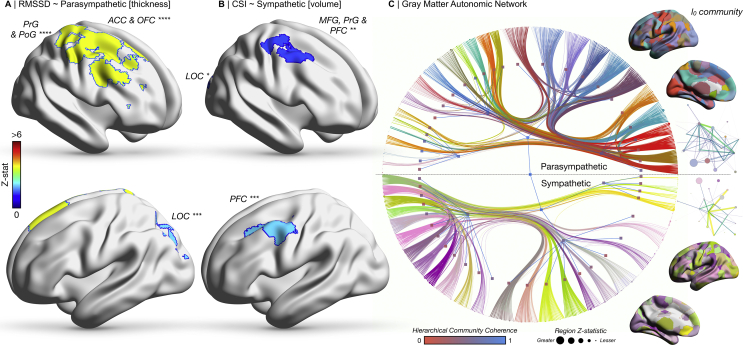
Brain gray matter structure and autonomic regulation. A) Cortical thickness significantly relates to RMSSD. B) Cortical volumetry significantly relates to CSI. C) A weighted stochastic block model identifies a hierarchical community-based gray-matter morphometric parcellation of brain regions implicated in the regulation of the ANS. Nodes size is proportional to z-statistic of voxel-based morphometry dependent on sympathetic and parasympathetic tone. Edge width is proportional to effect size of connections from network-based statistics. Hierarchical node colour is proportional to its coherence with the alternate autonomic contrast. A fully labelled high-resolution representation of this network is available as supplementary Fig. 2. Brain images on the right depict the level 0 (l_0_) community structure of the given autonomic model. Graphical representations on the far right of the figure illustrate the community network architecture of the parcellation at the given unit, where nodes are colour coded to the parcellation, sized proportionately to the number of regions they contain, with edge colour and width proportionate to the degree count between communities. Abbreviations: ACC, anterior cingulate cortex; OFC, orbitofrontal cortex; LOC, lateral occipital cortex; MFG, middle frontal gyrus; PFC, prefrontal cortex; PoG, postcentral gyrus; PrG, precentral gyrus.

We identified a large array of widely distributed gray matter areas modulated by resting autonomic tone. The extensive gray matter networks observed were dominated by regions within the frontal pole, orbitofrontal cortex, insula, diencephalon (thalamus), basal ganglia (caudate and putamen), hippocampus, amygdala and nucleus accumbens. Crucially, while under mass univariate analysis sympathetic and parasympathetic regions were similar, their graphical community structures differed radically (Fig. 2).

#### 3.3.1 Parasympathetic

Across the cortex, RMSSD was negatively correlated with cortical thickness in the right anterior cingulate, orbitofrontal cortex, precentral and postcentral gyrus (all *p* < .0001) and left lateral occipital cortex (*p* = .008). No significant positive correlations were identified, nor was an association to cortical volumetry. In the diencephalon, RMSSD was significantly associated with modulation of shape of the thalamus bilaterally (both *p* = .01) (Supplementary Figure 1). Network based statistics identified a gray-matter morphometric network of 187 nodes and 346 edges (*p* = .05), positively associated with RMSSD, encompassing both cortical and subcortical regions, including the frontal pole and orbitofrontal cortex (degree [number of significant adjoining edges] 105), cingulate (degree 39), insula (degree 22), caudate (degree 7), hippocampus (degree 6), putamen (degree 5), nucleus accumbens (degree 5) and thalamus (degree 2) (Supplementary Figure 2).

#### 3.3.2 Sympathetic

A negative correlation between CSI and cortical volume was observed in left prefrontal cortex (*p* = .008), right prefrontal cortex, precentral gyrus, middle frontal gyrus (all *p* = .01) and lateral occipital cortex (*p* = .04). No significant positive correlations were identified, nor was there an association with cortical thickness. Subcortical analyses showed significant modulation of shape at the right accumbens (*p* < .05) and right caudate (*p* = .04) by CSI (Supplementary Figure 1). Network based statistics identified a gray-matter network positively related to CSI of 75 nodes and edges (*p* = .01), including many edges connecting the middle frontal gyrus (degree 72), but also the cingulate (degree 8), hippocampus/hippocampal gyrus (degree 5), orbitofrontal cortex (degree 4), amygdala (degree 3), thalamus (degree 2), caudate, and accumbens (Supplementary Figure 2). Sympathovagal balance was also reflected in regional grey matter structure, reviewable in the supplementary material and Supplementary Figures 1 and 3.

Importantly, whilst we found no significant difference between the mass univariate effects of RMSSD and CSI on gray matter structure, their hierarchical community organization, revealed by the stochastic block model, differed radically. For the parasympathetic arm, we found that multiple insula, thalamic and anterior cingulate nodes formed communities together at the level 0 (l_0_) and level 1 (l_1_) hierarchical blocks, whilst other regions such as the amygdala, hippocampus, accumbens and posterior cingulate nodes would community together (Supplementary Figure 2). There were 27 l_0_ blocks and 4 l_1_ blocks derived from the parasympathetic contrast. In the sympathetic arm, regions such as the caudate, putamen and insula would community together, as would the insula, anterior cingulate, amygdala, accumbens, orbitofrontal cortex and hippocampi. The sympathetically derived communities were more equally sized, and largely contained both regions reported key in the central autonomic network (such as the cingulate), but also community to numerous other cortical regions, such as the pre- and post–central gyri. There were 25 l_0_ blocks and 3 l_1_ blocks in this sympathetic contrast.

### 3.4 White matter structure

**Fig. 3:**
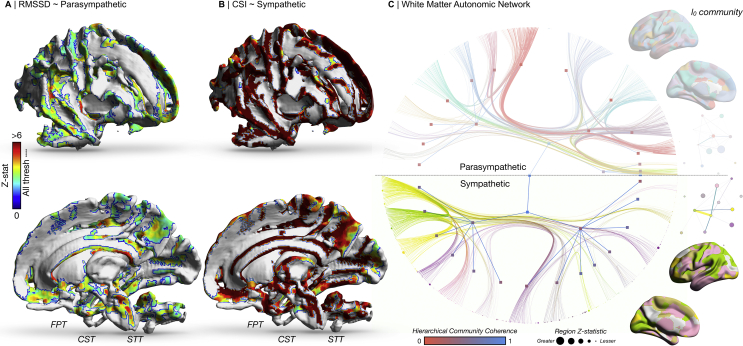
Brain white matter structure and autonomic regulation. A-B) Fractional anisotropy is significantly related to both RMSSD and CSI, with a preponderance for effect size at the corticospinal, spinothalamic tract and fronto-pontine tracts. C) A weighted stochastic block model identifies a hierarchical community-based probabilistic tractography parcellation of brain regions implicated in the regulation of the sympathetic nervous system (parasympathetic not significantly different after multiple comparisons in network-based statistics). Nodes size is proportional to z-statistic of fractional anisotropy related to sympathetic and parasympathetic tone. Edge width is proportional to effect size of connections from network-based statistics. Hierarchical node colour is proportional to its coherence with the alternate autonomic contrast. A fully labelled high-resolution representation of this network is available as supplementary Fig. 4. Brain images on the right depict the level 0 (l_0_) community structure of the given autonomic model. Graphical representations on the far right of the figure illustrate the community network architecture of the parcellation at the given unit, where nodes are colour coded to the parcellation, sized proportionately to the number of regions they contain, with edge colour and width proportionate to the degree count between communities. Abbreviations: CST, corticospinal tract; FPT, fronto-pontine tract; STT, spinothalamic tract.

We identified extensive differences throughout the brain white matter dependent on resting autonomic tone. Tract-based analysis showed that FA was positively correlated with CSI and RMSSD across multiple white matter areas, including corticospinal and spinothalamic tracts. Network analysis revealed a large sympathetic network, involving core regions such as the cingulate and orbitofrontal cortices, insula, subcortex (including numerous edges involving the hippocampus) and brainstem. Unlike its parasympathetic counterpart, the community structure of this network was significantly different from the null. Overall, the differences in the graphical structure between the two systems were more pronounced than FA (Fig. 3).

#### 3.4.1 Parasympathetic

Tract based spatial statistics identified many areas of the white matter skeleton whose FA was positively correlated with RMSSD (*p-*thresh<.0001). The white matter regions of highest effect size and significance included the corticospinal tract, spinothalamic tract, fronto-pontine tract, the medial lemniscus, the anterior thalamic radiations, corpus callosum and internal capsule (all bilateral). Analysis of axial, radial and mean diffusivity revealed similar anatomical patterns. Network based statistics did not yield a significant white matter network (Supplementary Figure 4).

#### 3.4.2 Sympathetic

The distribution of FA closely followed the parasympathetic map, with the tracts of highest effect size again including the corticospinal, spinothalamic, fronto-pontine, the medial lemniscus, the anterior thalamic radiations, corpus callosum and internal capsule (all bilateral) (*p-*thresh<.0001). Axial, radial and mean diffusivity were once again similar. The effect size for CSI was significantly greater than for RMSSD, though no regions were unique to either. Rather, there was a strong regional correlation between the local FA in the two conditions (R^2^ = .76, *p* < .0001) (Supplementary Figure 5). Network based statistics identified a white matter network negatively related to resting CSI, consisting of 109 nodes and 133 edges (*p* = .003). This included edges incorporating the hippocampus/parahippocampal gyrus (degree 30), frontal pole/orbitofrontal cortex (degree 29), cingulate (degree 22), insula (degree 8), putamen (degree 4), nucleus accumbens and brainstem (Supplementary Figure 4).

This network architecture and community allocation differed extensively between parasympathetic and sympathetic models (Supplementary Figure 4). The parasympathetic arm identified a hierarchical structure of 14 blocks at l_0_ and 3 at l_1,_ whilst the sympathetic branch in contrast consisted of 14 blocks at l_0_ and 2 at l_1_. For the sympathetic network community structure, brainstem, cingulate, accumbens, pre and post–central gyri nodes coalesced to a l_0_ block, as did insula with further cingulate nodes in another. In contrast, in the parasympathetic arm we found that several amygdala, accumbens, brainstem, insula, anterior and posterior cingulate nodes clustered together at an l_0_ block.

### 3.5 Resting function

**Fig. 4:**
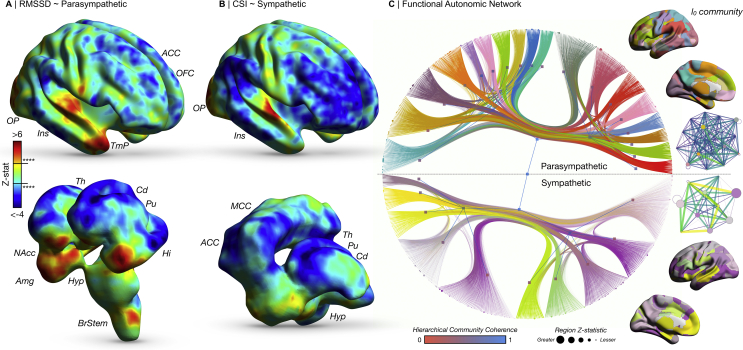
Brain function and autonomic regulation. A) Cortical and subcortical regions activity significantly related to both RMSSD and CSI (B). C) A weighted stochastic block model identifies a hierarchical community-based functional parcellation of brain regions implicated in the regulation of the ANS. Nodes size is proportional to z-statistic of resting activity related to sympathetic and parasympathetic tone. Edge width is proportional to effect size of functional connections from network-based statistics. Hierarchical node colour is proportional to its coherence with the alternate autonomic contrast. A fully labelled high-resolution representation of this network is available as supplementary Fig. 6. Brain images on the right depict the level 0 (l_0_) community structure of the given autonomic model. Graphical representations on the far right of the figure illustrate the community network architecture of the parcellation at the given unit, where nodes are colour coded to the parcellation, sized proportionately to the number of regions they contain, with edge colour and width proportionate to the degree count between communities. Abbreviations: ACC, anterior cingulate cortex; Amg, amygdala; ANS, autonomic nervous system; BrStem, brainstem; Cd, caudate; Hi, hippocampus; Hyp, hypothalamus; Ins, insula; MCC, mid cingulate cortex; NAcc, nucleus accumbens; OFC, orbitofrontal cortex; OP, occipital pole; Pu, putamen; Th, thalamus; TmP, temporal pole.

Multiple brain areas were modulated by resting autonomic tone. An extensive functional brain network modulated by both parasympathetic and sympathetic tone emerged, with a preponderance for frontal lobe regions including the orbitofrontal cortex, cingulate cortices, in addition to the caudate, putamen, thalamus, amygdala, nucleus accumbens and brainstem. In common with gray and white matter, while univariate effects were similar, the community structure of the sympathetic and parasympathetic graphical networks differed extensively (Fig. 4).

#### 3.5.1 Parasympathetic

RMSSD was significantly positively correlated with activity in the bilateral insula cortex, the bilateral temporal poles, orbitofrontal cortex, anterior cingulate cortex, the occipital pole, bilateral nucleus accumbens, bilateral amygdala, the hypothalamus, posterior brainstem structures, lingual gyrus, cuneus and precuneus. RMSSD was significantly negatively correlated to activity in the bilateral caudate, bilateral putamen, bilateral hippocampus and medial thalamic nuclei (all *p* < .0001). Network based statistics identified a functional brain network positively correlated to RMSSD of 216 nodes and 570 edges (*p* < .0001). This included edges between predominantly cortical regions, with many edges including the cingulate (degree 127), insula (degree 33) and orbital frontal cortex (degree 16) (Supplementary Figure 6). Brain regions in communities such as the default mode and cinguloparietal networks exhibited the greatest eigenvector centrality, a centrality measure of influence on the overall network structure), while those falling within the umbrella of the subcortex and salience networks were of significantly lower centrality, weighted by the RMSSD-derived edge statistics (*p* < .0001) (Supplementary Figure 7).

#### 3.5.2 Sympathetic

CSI was significantly positively correlated with activity in the bilateral insula cortex, occipital pole, cuneus and precuneus. CSI was significantly negatively correlated with activity in the anterior and mid cingulate cortex, medial thalamic nuclei, the bilateral caudate, the bilateral putamen and hypothalamus (all *p* < .0001). Network based statistics identified a functional brain network positively correlated to CSI of 95 nodes and 151 edges (*p* < .0001). This included edges between both cortical and subcortical regions, including the cingulate (degree 28), brainstem (degree 5), amygdala (degree 5), bilateral nucleus accumbens (degree 4), insula (degree 3), thalamus and putamen (Supplementary Figure 6). We showed a converse profile of centrality, wherein nodes of the subcortex, cinguloparietal and salience networks had significantly greater eigenvector centrality compared to those of the default mode network, weighted by CSI-derived edge statistics (*p* = .0002) (Supplementary Figure 7). CSI was weakly negatively correlated with small world propensity (r −.17, *p* = .001) and clustering coefficient (r −.14, *p* = .01). As with regional FA, the effect size and z-statistics of resting functional activity and its relationship to RMSSD and CSI were strongly positively correlated (R^2^ = .52, *p* < .0001) (Supplementary Figure 8). Findings pertaining to sympathovagal balance are presented in the supplementary material (Supplementary Figure 9).

Similar to the aforementioned gray matter and white matter network architecture, the community structure differed extensively between the parasympathetic and sympathetic resting functional connectivity stochastic block models (Supplementary Figure 6). The parasympathetic arm identified 18 l_0_ blocks and 2 at l_1_, whereas the sympathetic arm consisted of 10 blocks at l_0_ and 2 l_1_. As described above, the parasympathetic network invoked a large number of edges illustrating the vast interconnectedness of much of the brain with respect to resting parasympathetic tone. To that end, we found that brainstem, caudate, insula, thalamic, frontal pole, amygdala, globus pallidus and putamen nodes came together at a an l_0_ block, with a separate block consisting of multiple cingulate, pre- and post–central gyri and thalamic nodes. These separate communities would coalesce at the subsequent hierarchical level, the l_1_ block. Edge weights for the sympathetic arm were generally smaller, though we identified communities involving multiple orbitofrontal cortex, thalamic, hippocampal, putamen and insula nodes in one l_0_ block, with an adjacent l_0_ block consisting of multiple cingulate, accumbens, brainstem and amygdala nodes. These communities would then coalesce at the next hierarchical level.

### 3.6 Multi-modal, high-dimensional, generative, hierarchical autonomic connectomes

**Fig. 5:**
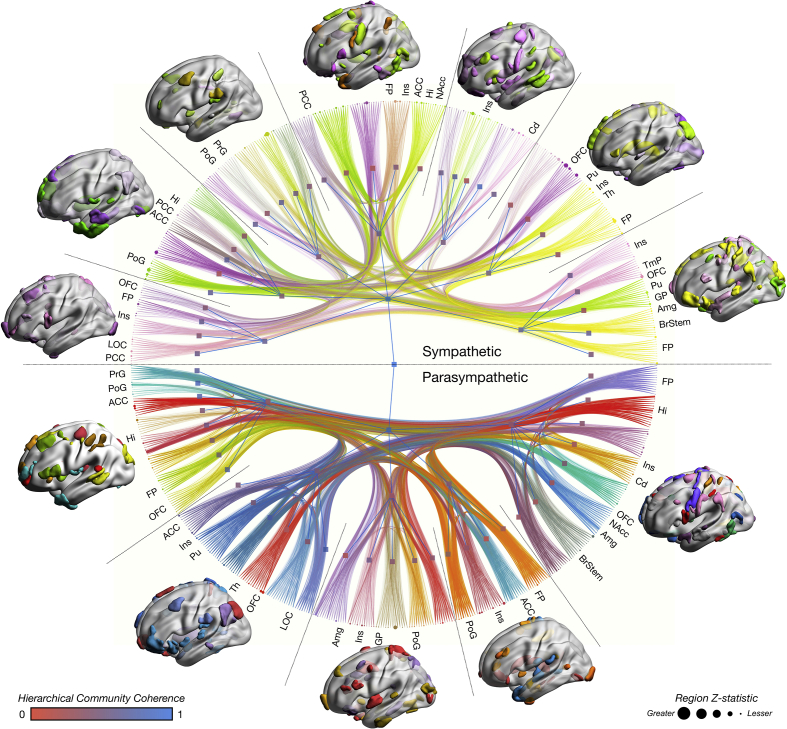
The parasympathetic and sympathetic connectome. A high-dimensional stochastic block model, weighted by network-based statistics derived from resting functional brain activity, gray matter-morphometry and white matter probabilistic tractography, identifies an intricate, hierarchical community-based parcellation implicated in parasympathetic and sympathetic regulation. Node sizes are proportional to summed z-statistics from functional, gray-matter morphometry and tract based spatial statistics. Edge width is proportional to summed network-based statistics. Hierarchical node colour is proportional to its coherence with the alternate autonomic contrast. Representative regions implicated in the parcellation are colour-coded according to the colour of the lowest level community of the hierarchical model. Select nodes are labelled for reference, though a full labelled network is available in supplementary Fig. 10. Abbreviations: ACC, anterior cingulate cortex; Amg, amygdala; BrStem, brainstem; Cd, caudate; FP, frontal pole; GP, globus pallidus; Hi, hippocampus; Ins, insula; NAcc, nucleus accumbens; LOC, lateral occipital cortex; OFC, orbitofrontal cortex; PCC, posterior cingulate cortex; PoG, postcentral gyrus; PrG, precentral gyrus; Pu, putamen; TmP, temporal pole.

We next combined all the preceding structural and functional analyses within a unified stochastic block model to generate a multi-modal, high-dimensional, weighted, non-parametric and hierarchical representation of the autonomic nervous system (Fig. 5). The edge weights of the model were significant 346 × 346 t-statistic adjacency matrices from all network-based analyses (RMSSD and CSI adjacency matrices, x 3 imaging sequences evaluated, 358,110 unique edge weights). First, we identified multimodal differences and similarities in the graphical community structure of the sympathetic and parasympathetic nervous systems, revealing a complex, intricate, hierarchical representation of the autonomic connectome (Fig. 5, high resolution fully labelled copy in Supplementary Figure 10). At the first order (l_0_) there were 64 communities, coalescing into 12 communities at the second order (l_1_) and finally into 2 at the third order (l_2_), corresponding to sympathetic and parasympathetic tone. Communities were dominated by structural and functional similarity, and naturally retained elements of organization seen within both the unimodal networks we defined, but also to that of networks already well characterized elsewhere, such as the default mode network. The inherently hierarchical nature of the organization was reinforced by a posterior odds ratio of Λ ≈ e^420913^ ≈ infinitely in favour of the hierarchical representation in place of the non-hierarchical alternative. To quantify the degree of difference and similarity, we evaluated the community coherence of brain regions across sympathetic and parasympathetic models by normalized mutual information (NMI), identifying that the architecture of these networks differs extensively. Mean NMI between RMSSD and CSI communities at l_0_ was .53 ± .15 (range .18–.64), whilst at l_1_ this was .49 ± .10 (range .30–.63). Labelled communities and NMI approximations are reviewable on Fig. 5 and Supplementary Figure 10.

**Fig. 6:**
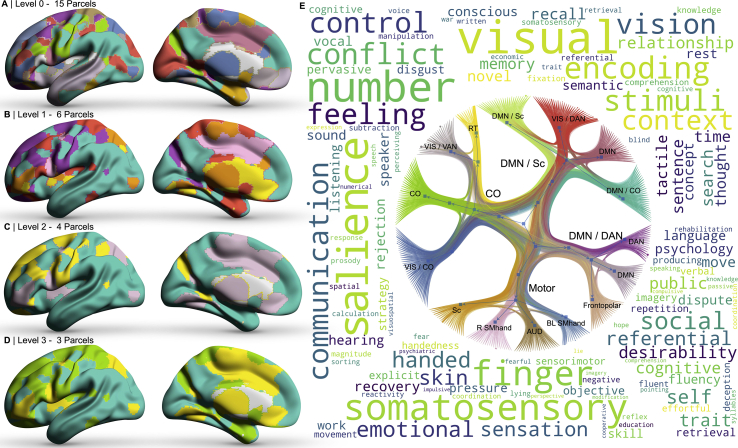
The autonomic connectome. A multi-modal, hierarchical parcellation scheme of autonomic nervous system function, defined by multi-modal graphical networks of both the parasympathetic and sympathetic nervous system. Hierarchical parcellation from level 0 (A – 15 parcels) through to level 3 (D – 3 parcels), wherein communities ultimately co-localise to a single unit. E) Graphical representations illustrate the community network architecture of the parcellation at the given unit, where nodes are colour coded to the parcellation. Around the graphical plot, we use word clouds to illustrate the weighted term frequency matches from NeuroQuery meta-analytic data with respect to the level 2 parcellation (panel C). Hierarchical nodes are labelled automatically by their closest fit to established community structures. Abbreviations: AUD, auditory control network; BL, bilateral; CO, cingulo-opercular network; DMN, default mode network; DAN, dorsal attention network; R, right; RT, retrosplenial-temporal community; SMhand, somatomotor hand system; Sc, subcortical regions; VAN, ventral attention network; VIS, visual network.

Lastly, we employed the nested stochastic block model to generate a hierarchical community structure of the brain representing autonomic tone overall. We achieved this by use of all imaging data reported, including the contrast edge weights of gray, white matter and functional network data across both sympathetic and parasympathetic contrasts. This identified a 4-level hierarchical parcellation scheme of the brain with respect to its multi-modality graphical association of both sympathetic and parasympathetic tone. At its lowest level (l_0_), a 15 regional parcellation was delineated, which featured organised communities of motor, auditory, dorsal attention, default mode, frontopolar, cingulo-opercular, retrosplenial-temporal, visual, ventral attention and subcortex. This hierarchically converged to 6 regions at l_1_, 4 regions at l_2_, organised into communities of motor, default mode/dorsal attention, default mode/subcortex and cingulo-opercular, and 3 regions at l_3_ (wherein the subsequent hierarchical level, l_4_, would simply encompass the whole brain). We illustrate the hierarchical parcellation and its community structure in Fig. 6, making it available in NIFTI and tabulated format in the supplementary data. We compared the underlying community structure to that of the retrieved meta-analytic corpus at the l_2_ level. This revealed organisation of domains of the following: i) sensorimotor; ii) visuospatial; iii) cognitive-evaluative and iv) social reflective).

## 4 Discussion

In the largest and most comprehensively studied group of individuals to date—incorporating the relationship between cortical, tractographic, functional brain mapping and heart rate variability—we reveal in unprecedented detail the rich structural and functional brain signature of the autonomic nervous system. We provide a generative, hierarchical, graphical model of the brains architecture underpinning the autonomic nervous system as disclosed by heart rate variability, an approach unifying all available modalities, drawn from a large group of individuals of all ages encompassing the full spectrum of autonomic normality and abnormality. The complex brain networks thereby revealed plausibly underpin autonomic functions that covary with heart rate variability: in describing them so richly, we acquire potentially greater fidelity in characterising pathological deviations across the full range of diseases with impact on autonomic function. Our analysis provides a graphical network schema for evaluating autonomic disruption at a variable, hierarchical granularity that can be tailored to the statistical power of a specific study.

### 4.1 Brain gray matter structure and autonomic regulation

We show how gray matter structure is related to autonomic regulation at cortical regions inclusive of the anterior cingulate, orbitofrontal and prefrontal cortices. We identify regions whose shapes differ contingent on autonomic tone, including the thalamus, nucleus accumbens and basal ganglia. Our univariate findings at both the cortex and subcortex reproduce those of other published studies (Ruffle, Coen, Giampietro, Williams, Apkarian, et al., 2018), including a large multi-site study of brain structure (Koenig, Abler, Agartz, et al., 2021). We advance current knowledge in identifying gray matter morphometric networks (Raamana & Strother, 2018) subserving autonomic regulation, with high-degree nodes including the orbitofrontal cortex, cingulate, insula, basal ganglia, thalamus, nucleus accumbens, amygdala and hippocampus. While our univariate findings replicate published studies, our graphical analysis now uniquely reveals their inter-relations, describing not just the network, but the community structure within it.

### 4.2 Brain white matter structure and autonomic regulation

The white matter connectivity of the autonomic system in normal health has received little attention, most published studies focusing on the impact of major pathology such as Parkinson's disease (Ashraf-Ganjouei, Majd, Javinani, & Aarabi, 2018) or stroke (Williamson et al., 2012). In consequence, the relationship between autonomic tone and the characteristics of white matter tracts have until now been unknown. Here, we identify the clear relationship between resting autonomic tone and many areas of the white matter skeleton, with focus on the spinothalamic, corticospinal and fronto-pontine tracts. With tractography, we illustrate a sympathetic-specific white matter network structurally linking many autonomically salient gray matter regions, including orbitofrontal cortex, cingulate, insula, hippocampus, nucleus accumbens and brainstem. These findings reveal the importance of white matter ascending tracts, brainstem, subcortical and cortical regions with regard to central autonomic regulation (Critchley & Harrison, 2013).

### 4.3 Brain function and autonomic regulation

We reveal a complex hierarchical network of functional brain connectivity associated with autonomic tone, implicating a wide array of cortical and subcortical regions, with clear differences between sympathetic and parasympathetic systems. We further show that the activity of key cortical and subcortical regions (including multiple regions within the default mode network) are implicated in autonomic regulation, including the anterior and mid cingulate cortices, orbitofrontal cortex, insula, thalamus, amygdala, nucleus accumbens, hypothalamus, hippocampus, basal ganglia and brainstem structures. These findings consolidate previous studies on smaller individuals/pooled meta-analyses (Beissner et al., 2013; Ruffle, Coen, Giampietro, Williams, Aziz, et al., 2018), and reinforce the argument that the default mode network may serve an overarching regulatory role across a variety of human physiology and behaviour (Dohmatob, Dumas, & Bzdok, 2020).

### 4.4 The autonomic brain: multidimensional generative hierarchical modelling of the autonomic connectome

Autonomic neuroimaging studies have to date mostly focused on evaluating the relationship between autonomic function and a single imaging modality or brain characteristic, omitting the multimodal level of organisation of the brain. Given the central importance of the autonomic nervous system, and its frequent disturbance in disorders spanning multiple body systems, we argue its operations are bound to reflect the complex interplay of many areas of the brain, best captured as a graph. To arrive at the closest approximation to the underlying true organisation should therefore require a multi-dimensional generative graphical model that comprehensively integrates structure and function across the brain. Our approach of using a Bayesian weighted and hierarchical stochastic block model—a generative graphical model—to identify a hierarchical community of brain regions implicated in parasympathetic and sympathetic regulation is here shown to be strongly favoured over a non-hierarchical alternative. The resultant multi-dimensional ‘autonomic connectome’ reveals the complex hierarchical interplay between cortical and subcortical communities in autonomic regulation, yielding a hierarchical parcellation of the substrate plausibly involved in autonomic regulation and its deviation from normality, providing a means to utilise this in future imaging study. Meta-analytic functional labelling suggests a high-level organisation into sensorimotor, visuospatial, cognitive-evaluative and social-reflective domains.

### 4.5 The value of graphical modelling

Our analysis attests to the value of the network approach to brain imaging (Bullmore & Sporns, 2009; Rubinov & Sporns, 2010; Sporns, 2013). We have demonstrated that the mass-univariate evaluation of functional activity, white matter architecture, and gray matter morphometry robustly identifies regions modulated by *both* parasympathetic and sympathetic tone, but not regions that *differ* between the two systems. Yet, the corresponding graphical networks are radically different. This indicates that the regions implicated in autonomic control are unlikely to be uniquely allocated to regulating sympathetic or parasympathetic function, but rather interact in distinctive ways in subserving each function. To understand the operation of the autonomic system as a whole we argue it is the network level we must interrogate.

We have further shown that graphical analysis can reveal communities of multiple neural areas sharing similar patterns of inter-relatedness that may have mechanistic implications for the organisation of autonomic function. The communities identified here are potential targets for more detailed examination with correlative and especially with disruptive techniques, illuminating the significance of the observed distinct patterns of shared connectivity. Such downstream investigation is facilitated here by employing stochastic block models to derive a hierarchical parcellation of the brain into regions-of-interest optimally tuned to detecting differences between autonomic areas. A system-specific, hierarchical parcellation provides a flexible means of parameterising the brain at a granularity chosen such that the functional anatomical comparability of a set of individuals under study is optimised for the available data. Where only a few individuals are available, a coarser parcellation, obtained from higher levels of the hierarchy would be appropriate, and the converse will be true where a great deal of data is available. But whatever the chosen scale, the comparability of each individual on the dimensions that matter—those related to autonomic function—would be superior to a parcellation of the same resolution that is not informed by the system under study. Our models also provide a principled mechanism for integrating multimodal information in quantifying the impact of regional neural dysfunction on any individual patient. For example, a focal lesion such as an ischaemic stroke can now be weighted—separately for grey and white matter components—to produce a synoptic index of potential autonomic impact, compactly expressed. Such “functionally-informed” multimodal lesion representations can facilitate predictive models of individual clinical outcomes, and enhance the detection of individual therapeutic effects (Xu, Rolf Jäger, Husain, Rees, & Nachev, 2017). Finally, the same generative graphical approach can be extended to autonomic measures themselves, extracting a hierarchically organised representation of their relations that can then be used to provide an even richer description of the autonomic brain, constrained only by the scale and diversity of available data.

### 4.6 Strengths and limitations

All models of the brain are necessarily approximations: our aim is less to produce the definitive map of the autonomic nervous system than to provide a blueprint for generating iteratively refined multimodal maps that grow in detail and robustness with the addition of data, encompassing new investigational modalities and broader populations. It would be valuable to evaluate the population effects of parameters known to modulate autonomic activity, such as caffeine (Niedermaier et al., 1993) and smoking (Zahn & Rapoport, 1987), which our data did not permit us to examine or control for. Though our cohort deliberately excluded any substantive ill-health or medication use so as to allow us to focus on the spectrum of autonomic normality and abnormality only, these aspects could naturally be addressed in the same way, aided by the comparison of the present population. In all modelling, we controlled for all available demographic and baseline cardiovascular health parameters (including blood pressure), but we could not control for the time interval between autonomic testing and imaging owing to the nature of the prospective study. However, given an individual's autonomic signature has been shown as comparable over a year later (Farmer et al., 2014), it would seem plausible that the effect of this is limited. Future work should additionally validate these findings across other alternate surrogate measures of autonomic nervous system activity, including with real-time synchronous fMRI and HRV data. Replication on large-scale datasets diversified both biologically and instrumentally—such as the Human Connectome Project or UK Biobank (Alfaro-Almagro et al., 2018; Van Essen et al., 2013)—will strengthen generalisability. That all individuals here were studied and scanned on a single site scanner with the same sequence throughout, a rarity in large brain imaging cohorts.

## 5 Conclusion

We present the largest, most comprehensively studied group of individuals with respect to the central brain regulation of the autonomic nervous system and provide a hierarchical representation of a functionally defined physiological domain of the brain. Our multidimensional, generative hierarchical network of the central regulation of autonomic nervous system function reveals the topology of this complex and intricate system. Future studies should investigate this network with regards to its perturbation across major disease states.

## CRedit author statement

JKR: conceptualization, methodology, software, validation, formal analysis, writing – original draft, writing - review & editing, visualisation.

HH: resources, writing - review & editing.

MAH: resources, writing - review & editing.

ADF: resources, writing - review & editing.

AVA: resources, methodology, writing - review & editing.

SCRW: resources, writing - review & editing.

QA: conceptualisation, methodology, supervision, writing – original draft, writing - review & editing.

PN: conceptualisation, methodology, resources, supervision, writing – original draft, writing - review & editing.

## Funding

JR was supported by 10.13039/100014899American Neurogastroenterology and Motility Society, the CDT i4health and the Issac Schapero research grant.

PN is supported by the 10.13039/100010269Wellcome Trust (213038/Z/18/Z) and the 10.13039/501100008721UCLH 10.13039/501100000272NIHR 10.13039/100014461Biomedical Research Centre.

HH is supported by the 10.13039/501100008721UCLH 10.13039/501100000272NIHR 10.13039/100014461Biomedical Research Centre.

SCRW and MAH are supported by the 10.13039/501100000272National Institute for Health Research (NIHR) Biomedical Research Centre at the 10.13039/100009362South London and Maudsley NHS Foundation Trust and 10.13039/100009360King’s College London and the 10.13039/501100000265Medical Research Council (MR/N026969/1).

## Open practices

The study in this article earned an Open Data – Protected Access badge for transparent practices. All software and code used is freely available via the original referenced sources in the methods. Code excerpts are provided at https://osf.io/s3nej/.
