# Supplementary material for: The autonomic brain: Multi-dimensional generative hierarchical modelling of the autonomic connectome
Source: Cortex. 2021 Oct;143:164–79. doi: 10.1016/j.cortex.2021.06.012 (PMC8500219; doi:10.1016/j.cortex.2021.06.012)
Supplement: Multimedia component 13 [file mmc3.pdf]

FULL TITLE
The autonomic brain: multi-dimensional generative hierarchical modelling of the autonomic connectome

RUNNING TITLE
The autonomic brain

ARTICLE TYPE
Supplementary materials.

SUPPLEMENTARY METHODS

Derivation of autonomic measures

The following cardio-autonomic measures were calculated: 1) root mean square of successive RR interval differences (RMSSD), used as a surrogate to parasympathetic tone; 2) cardiac sympathetic index (CSI), used as a surrogate to sympathetic tone; and thirdly the ratio between the two used as a surrogate to sympathovagal balance. Some research groups use the LF:HF ratio as marker for sympathovagal balance; however, there has been growing concern this is erroneous(Billman, 2013). We thus felt it justified to manually model the ratio between the parasympathetic and sympathetic measures we specifically extracted and analysed, which would otherwise result in a rank-deficient general linear model, as well as potentially invalidate results.

Pre-processing and statistical analysis of neuroimaging data

Cortex-specific measures | FreeSurfer

In brief, this processing includes motion correction and averaging of multiple T1-weighted images; removal of non-brain tissue with the hybrid-watershed and surface deformation procedure; automated Talairach transformation; segmentation of subcortical white matter and deep gray matter volumetric structures; intensity normalisation; tessellation of the gray—white matter boundary; and automated topology correction and surface deformation post-intensity gradients to optimally place the gray/white and gray/CSF borders where the location of the greatest shift in intensity defines the transition to the other tissue class. After cortical models are complete, numerous deformable procedures can be performed for further data processing and analysis, including surface inflation, registration to a spherical atlas based upon individual cortical folding patterns to match cortical geometry across subjects, parcellation of cerebral cortex into units with respect to gyral and sulcal structure, and creation of a variety of surface-

based data, including curvature and sulcal depth maps. This method employed both intensity and continuity information from the entire 3D MR volume in segmentation and deformation procedures to produce representations of cortical thickness, calculated as the closest distance from the gray/white boundary to the gray/CSF boundary at each vertex on the tessellated surface. The maps are created using spatial intensity gradients across tissue classes and are therefore not fully reliant on absolute signal intensity. Maps produced are not restricted to voxel resolution of the original data, thus can detect submillimetre differences between groups. Procedures for measurement of cortical thickness have been validated against histological analysis and manual measurement. Statistical analysis was to investigate the left and right cortical hemispheres with regards to cortical thickness and cortical volumes.

Subcortical-specific measures | FSL FIRST

Vertex and volumetric analyses were performed using FSL-FIRST 5.0, a Bayesian modelling toolkit developed for segmentation, shape and volumetric analysis of the subcortex(Patenaude, Smith, Kennedy, & Jenkinson, 2011). This package firstly skull-strips with the FSL brain extraction tool (BET), following which performs segmentation of each patient structural scan into 15 subcortical structures: bilateral nucleus accumbens, amygdala, caudate, hippocampus, pallidum, putamen and thalamus, as well as the brainstem. Segmented structures are then registered to the Montreal Neurological Institute (MNI) 152 1mm template. The morphometry (shape deformations) of the subcortex were statistically tested. Subcortical volumes were extracted using FSL-STATS.

Voxel-based morphometry

Voxel-based morphometry were used as a pre-processing step to generate gray matter morphometric networks. This were undertaken using the CAT-12 (http://www.neuro.uni-

jena.de/cat/) adaptation of VBM within SPM12(Ashburner & Friston, 2005) for generation of gray-matter morphometric networks(Ramamana & Strother, 2018).

Network-based statistics

Network-based statistic (NBS) is a non-parametric statistical method which corrects for multiple comparisons and controls for family-wise error rate (FWE). The NBS is a graph analogue of cluster-based statistical methods used in mass-univariate testing on all voxels in an image and produces clusters in topological space, as opposed to physical space. The NBS relies on permutation testing (Freedman & Lane method(Freedman & Lane, 1983)) to determine significance within the GLM, which includes regression of nuisance predictors, permuting resulting residuals and subsequently adding permuted residuals back to nuisance signal to give a realisation of data under the null hypothesis. This approach recognizes that permuting raw data is not desirable as it may engender some variability explained by nuisance predictors. Rather, it is the error terms that can be permuted and estimated under the null hypothesis as a part of the data not explained by the nuisance regressors; that is, the residuals(Anderson & Robinson, 2008). The method permits derivation of FWER-corrected p values using permutation testing when investigating brain networks(Sporns, Tononi, & Kötter, 2005).

SUPPLEMENTARY RESULTS
Brain gray matter structure

Sympathovagal balance

We identified a significant positive correlation of cortical thickness to the RMSSD/CSI ratio involving the anterior cingulate gyrus, paracingulate and orbitofrontal cortex, insula, and precentral gyrus (all p=0.0002), inferior frontal gyrus and superior frontal gyrus (all p=0.007), angular gyrus, supramarginal gyrus, lateral occipital cortex and middle temporal gyrus (all

p=0.02). On the left hemisphere, we identified a negative correlation of cortical thickness to RMSSD/CSI ratio involving the lateral occipital cortex (*p*=0.02) (Supplementary Figure 3). No significant relationships to cortical volume were identified. Subcortical analysis identified shape changes at the left accumbens *(p*=0.04), right caudate (*p*=0.03) and left thalamus (*p*=0.03), contingent on the RMSSD/CSI ratio (Supplementary Figure 1). Network based statistics identified a gray-matter morphometric network negatively related to RMSSD/CSI, consisting of 87 nodes and 100 edges (*p*=0.04), including the frontal pole/orbitofrontal cortex (degree 45), insula (degree 6), cingulate (degree 6) and caudate nucleus (degree 5).

Brain white matter structure

Sympathovagal balance

With tract based spatial statistics, there were no significant differences in white matter skeleton fractional anisotropy associated to the RMSSD/CSI ratio. Similarly, network-based statistics did not identify a network specific to sympathovagal balance after correction for multiple comparisons.

Brain function

Sympathovagal balance

RMSSD/CSI was positively correlated to activity on the anterior, mid, and posterior cingulate cortex, hypothalamus, right nucleus accumbens and right orbitofrontal cortex (all $p<0.0001$) (Supplementary Figure 9). Network based statistics identified a small functional network negatively correlated to RMSSD/CSI of 23 nodes and 22 edges (*p*=0.039). This included several edges incorporating the cingulate cortex (degree 6).

SUPPLEMENTARY FIGURE LEGENDS

Supplementary Figure 1: Shape deformation of the subcortex significantly relates to resting autonomic function. Significant subcortical regions are displayed in green, with specific surfaces of significant deformation relating to CSI (red), RMSSD (blue) and RMSSD/CSI (yellow).

Supplementary Figure 2: Weighted stochastic block model of brain gray matter morphometry and autonomic processing. High resolution model result with regions of the parcellation labelled. The model is subdivided into regulation of parasympathetic and sympathetic tone. Node size is proportional to z-statistic of voxel-based morphometry related to sympathetic and parasympathetic tone. Edge width is proportional to effect size of connections from network-based statistics. Hierarchical node colour is proportional to its coherence with the alternate autonomic contrast. Abbreviations of brain regions is as per standard convention, though a label matrix is further provided as supplementary data.

Supplementary Figure 3: Cortical thickness relates to sympathovagal balance. Abbreviations: ACC, anterior cingulate cortex; Ins, insula; OFC, orbitofrontal cortex; PrG, precentral gyrus.

Supplementary Figure 4: A weighted stochastic block model identifies a hierarchical community-based probabilistic tractography parcellation of brain regions implicated in the regulation of the sympathetic nervous system (parasympathetic not significantly after multiple comparisons in network-based statistics). High resolution model result with regions of the parcellation labelled. Node size is proportional to z-statistic of fractional anisotropy related to sympathetic and parasympathetic tone. Edge width is proportional to effect size of connections from network-based statistics. Hierarchical node colour is proportional to its coherence with

the alternate autonomic contrast. Abbreviations of brain regions is as per standard convention, though a label matrix is further provided as supplementary data.

Supplementary Figure 5: Regulation of parasympathetic and sympathetic tone is significantly interrelated by white matter fractional anisotropy (FA). Z-statistics of the parcellation with respect to their association between FA to both CSI (x-axis) and RMSSD (y-axis) illustrates the two are significantly interrelated. Abbreviations: AUD, auditory control network; CO, cingulo-opercular network; CP, cingulo-parietal network; DMN, default mode network; DAN, dorsal attention network; FP, fronto-parietal network; RT, retrosplenial-temporal community; SAL, salience network; SMhand, somatomotor hand system; SMmouth, somatomotor mouth system; Sc, subcortical regions; VAN, ventral attention network; VIS, visual network.

Supplementary Figure 6: Weighted stochastic block model of brain function and autonomic processing. High resolution model result with regions of the parcellation labelled. The model is subdivided into regulation of parasympathetic and sympathetic tone. Node size is proportional to z-statistic of resting activity related to sympathetic and parasympathetic tone. Edge width is proportional to effect size of functional connections from network-based statistics. Hierarchical node colour is proportional to its coherence with the alternate autonomic contrast. Abbreviations of brain regions is as per standard convention, though a label matrix is further provided as supplementary data.

Supplementary Figure 7: Eigenvector centrality of communities weighted by parasympathetic and sympathetic edge weights. A) Violin plots of eigenvector centrality of brain regions when weighted by RMSSD, organized by brain community. For example, centrality of DMN nodes were significantly higher than the SAL and Sc communities. B) In

contrast, CSI-weighted eigenvector centrality of the CP, Sc and SAL network were significantly greater to that of the DMN. Abbreviations: AUD, auditory control network; CO, cingulo-opercular network; CP, cingulo-parietal network; DMN, default mode network; DAN, dorsal attention network; FP, fronto-parietal network; RT, retrosplenial-temporal community; SAL, salience network; SMhand, somatomotor hand system; SMmouth, somatomotor mouth system; Sc, subcortical regions; VAN, ventral attention network; VIS, visual network.

Supplementary Figure 8: Regulation of parasympathetic and sympathetic tone is significantly interrelated by brain function. Z-statistics of the parcellation with respect to their association between resting functional activity to both CSI (x-axis) and RMSSD (y-axis) illustrates the two are significantly interrelated. Abbreviations: AUD, auditory control network; CO, cingulo-opercular network; CP, cingulo-parietal network; DMN, default mode network; DAN, dorsal attention network; FP, fronto-parietal network; RT, retrosplenial-temporal community; SAL, salience network; SMhand, somatomotor hand system; SMmouth, somatomotor mouth system; Sc, subcortical regions; VAN, ventral attention network; VIS, visual network.

Supplementary Figure 9: Resting brain function and sympathovagal balance. Cortical and subcortical regions whose activity were significantly related to RMSSD/CSI. Abbreviations: ACC, anterior cingulate cortex; Hyp, hypothalamus; MCC, middle cingulate cortex; NAcc, nucleus accumbens; PCC, posterior cingulate cortex.

Supplementary Figure 10: High resolution labelled copy of the divergent parasympathetic-sympathetic connectome. A multidimensional stochastic block model, weighted by network-based statistics identified from resting functional brain activity, gray matter-morphometry and white matter probabilistic tractography identifies an intricate, hierarchical community-based

parcellation implicated in parasympathetic and sympathetic regulation. Node sizes are proportional to summed z-statistics from functional, gray-matter morphometry and tract based spatial statistics. Edge width is proportional to summed network-based statistics. Hierarchical node colour is proportional to its coherence with the alternate autonomic contrast. Representative regions implicated in the parcellation are colour-coded according to the colour of the lowest level community of the hierarchical model.

Supplementary Movie 1: Dynamic representation of generating the multi-modal, high-dimensional autonomic connectome. Node colour and size is proportionate to the effect size of its involvement in autonomic nervous system function. Edge colour and width is proportionate to the sum of its effect size across both gray matter, tractography and functional brain networks. Individual frames (60fps) correspond to single iterations of the model. Note how regions which are 'more connected', i.e., those with wider and brighter yellow edges, will begin to move closer together yet those which are 'less connected', i.e., those with thinner more purple edges, will be pushed away from the centre of the graph as the procedure iterates.

REFERENCES

Anderson, M. J., & Robinson, J. (2008). Permutation Tests for Linear Models. *Australian & New Zealand Journal of Statistics, 43*(1), 75-88. doi: 10.1111/1467-842X.00156

Ashburner, J., & Friston, K. J. (2005). Unified segmentation. *Neuroimage, 26*(3), 839-851. doi: 10.1016/j.neuroimage.2005.02.018

Billman, G. E. (2013). The LF/HF ratio does not accurately measure cardiac sympatho-vagal balance. *Front Physiol, 4*, 26. doi: 10.3389/fphys.2013.00026

Freedman, D., & Lane, D. (1983). A Nonstochastic Interpretation of Reported Significance Levels. *Journal of Business & Economic Statistics, 1*(4), 292-298. doi: 10.2307/1391660

Patenaude, B., Smith, S. M., Kennedy, D. N., & Jenkinson, M. (2011). A Bayesian model of shape and appearance for subcortical brain segmentation. *Neuroimage, 56*(3), 907-922. doi: 10.1016/j.neuroimage.2011.02.046

Ramamana, P. R., & Strother, S. C. (2018). graynet: single-subject morphometric networks for neuroscience connectivity applications. . *Journal of Open Source Software, 3*(30), 924.

Sporns, O., Tononi, G., & Kötter, R. (2005). The Human Connectome: A Structural Description of the Human Brain. *PLOS Computational Biology, 1*(4), e42. doi: 10.1371/journal.pcbi.0010042
