# Supplementary figures and images for: The autonomic brain: Multi-dimensional generative hierarchical modelling of the autonomic connectome
Source: Cortex. 2021 Oct;143:164–79. doi: 10.1016/j.cortex.2021.06.012 (PMC8500219; doi:10.1016/j.cortex.2021.06.012)

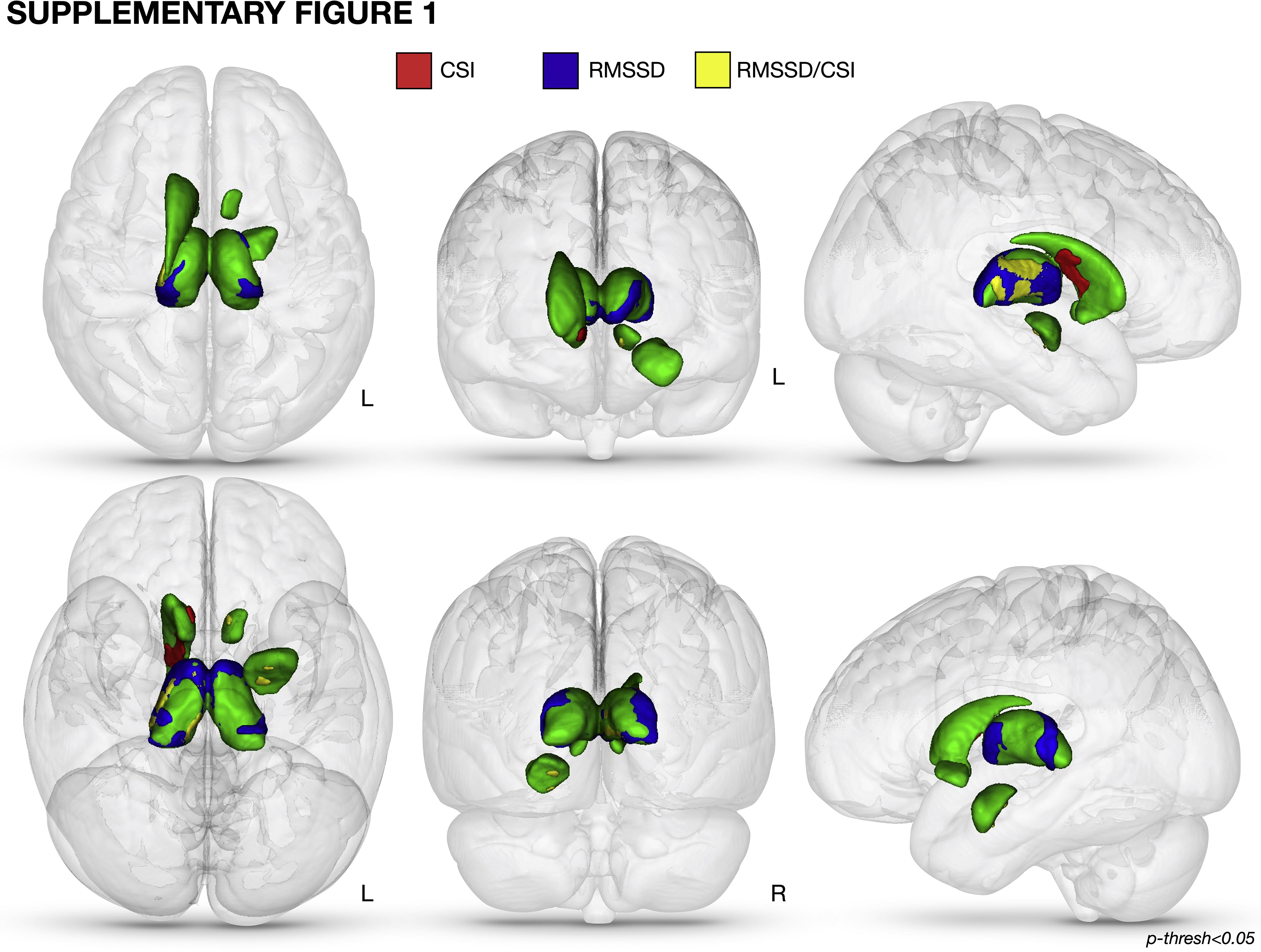

Supplement: Multimedia component 2 [file figs1.jpg]

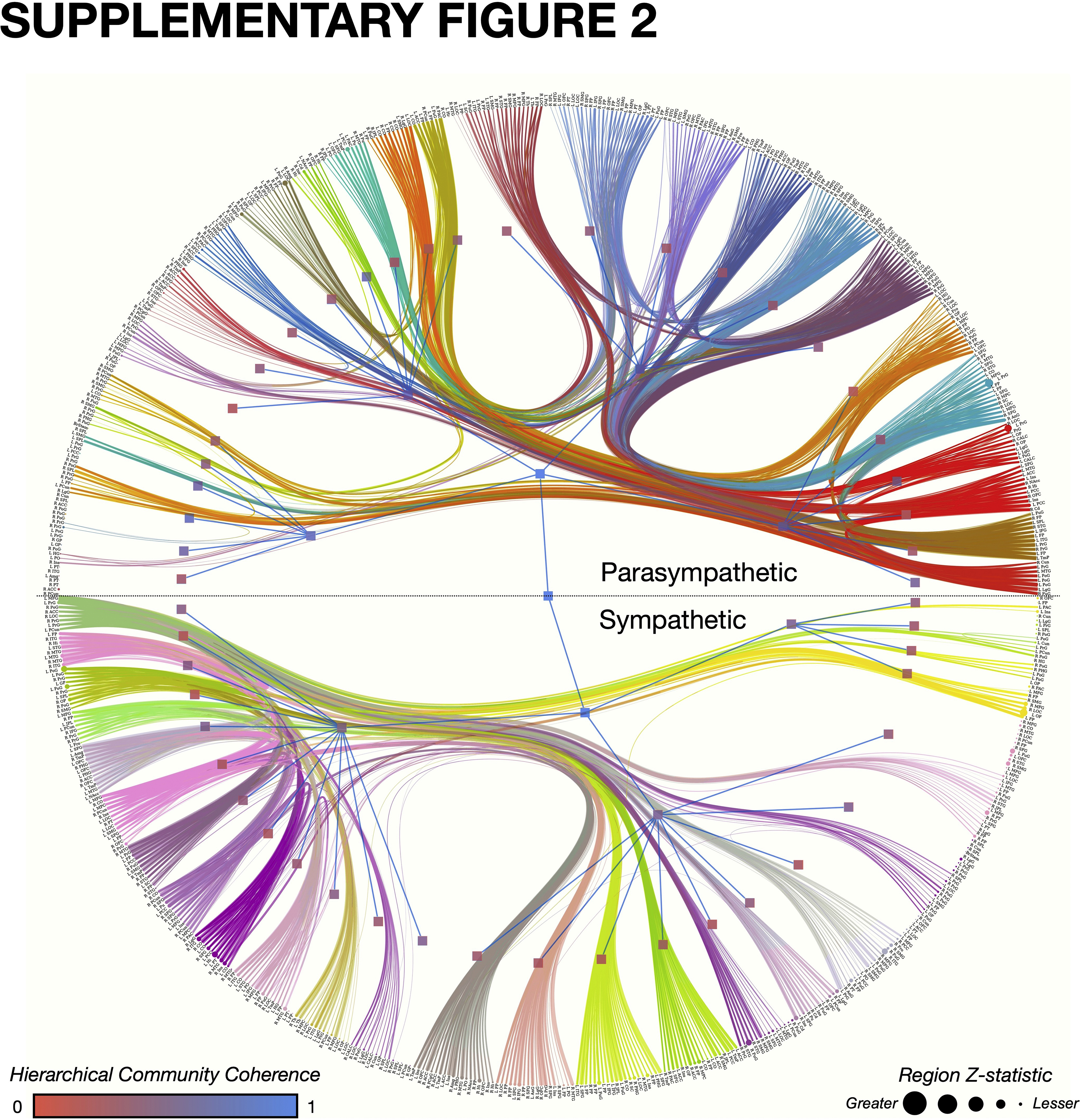

Supplement: Multimedia component 2 [file figs2.jpg]

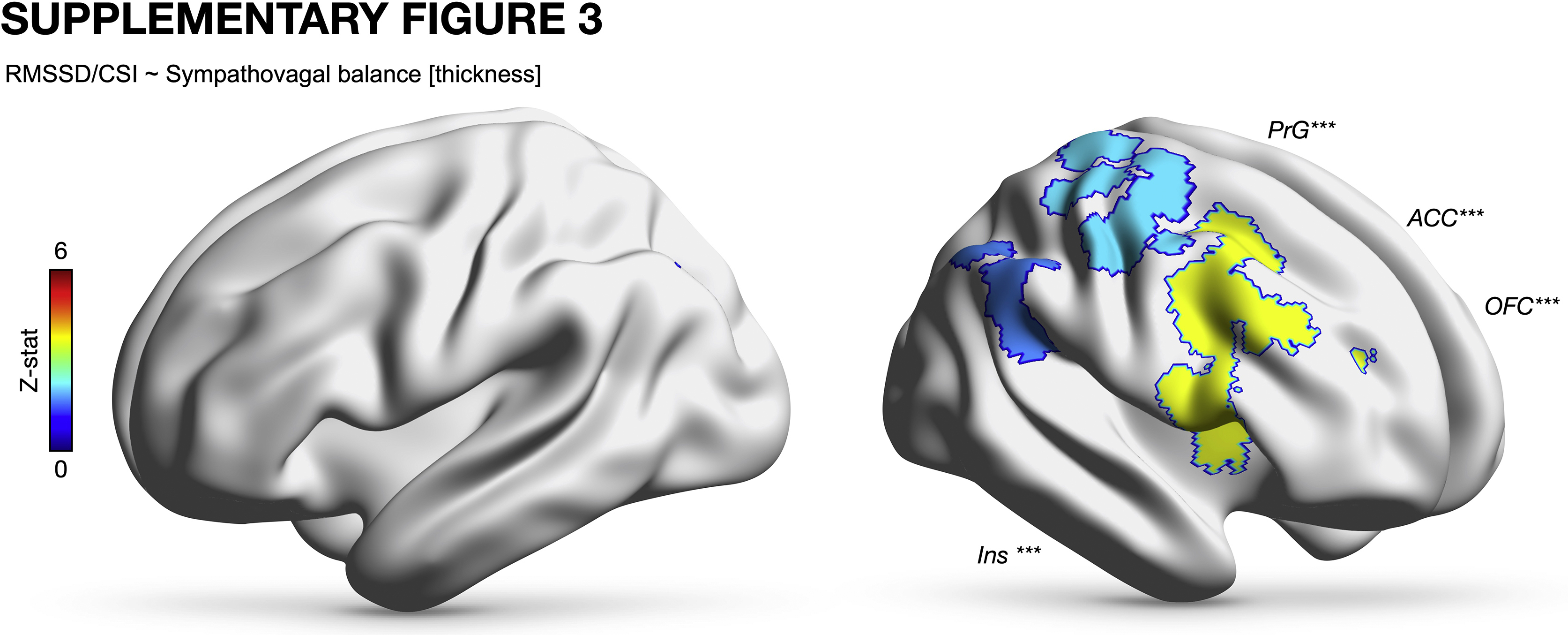

Supplement: Multimedia component 3 [file figs3.jpg]

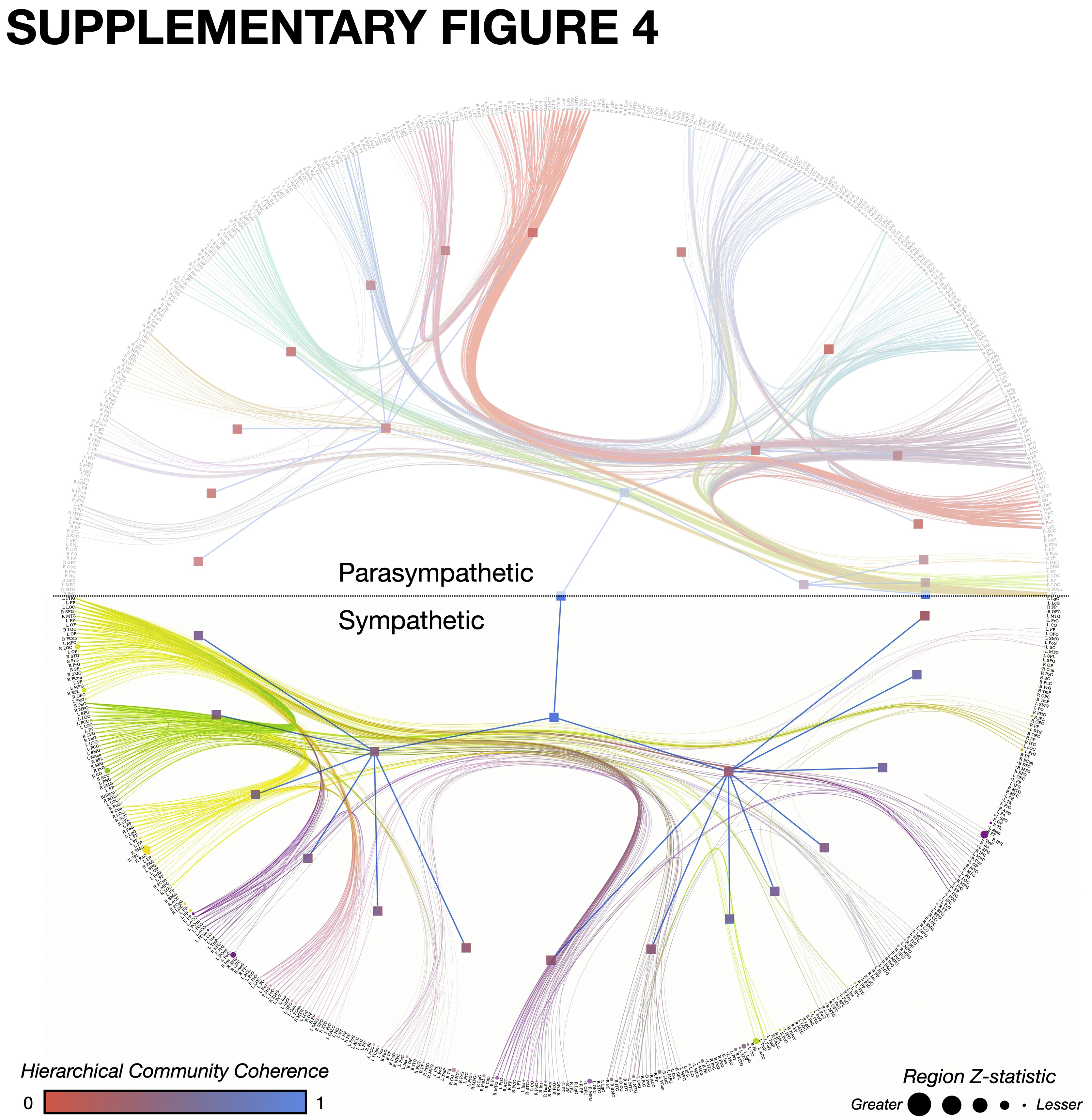

Supplement: Multimedia component 4 [file figs4.jpg]

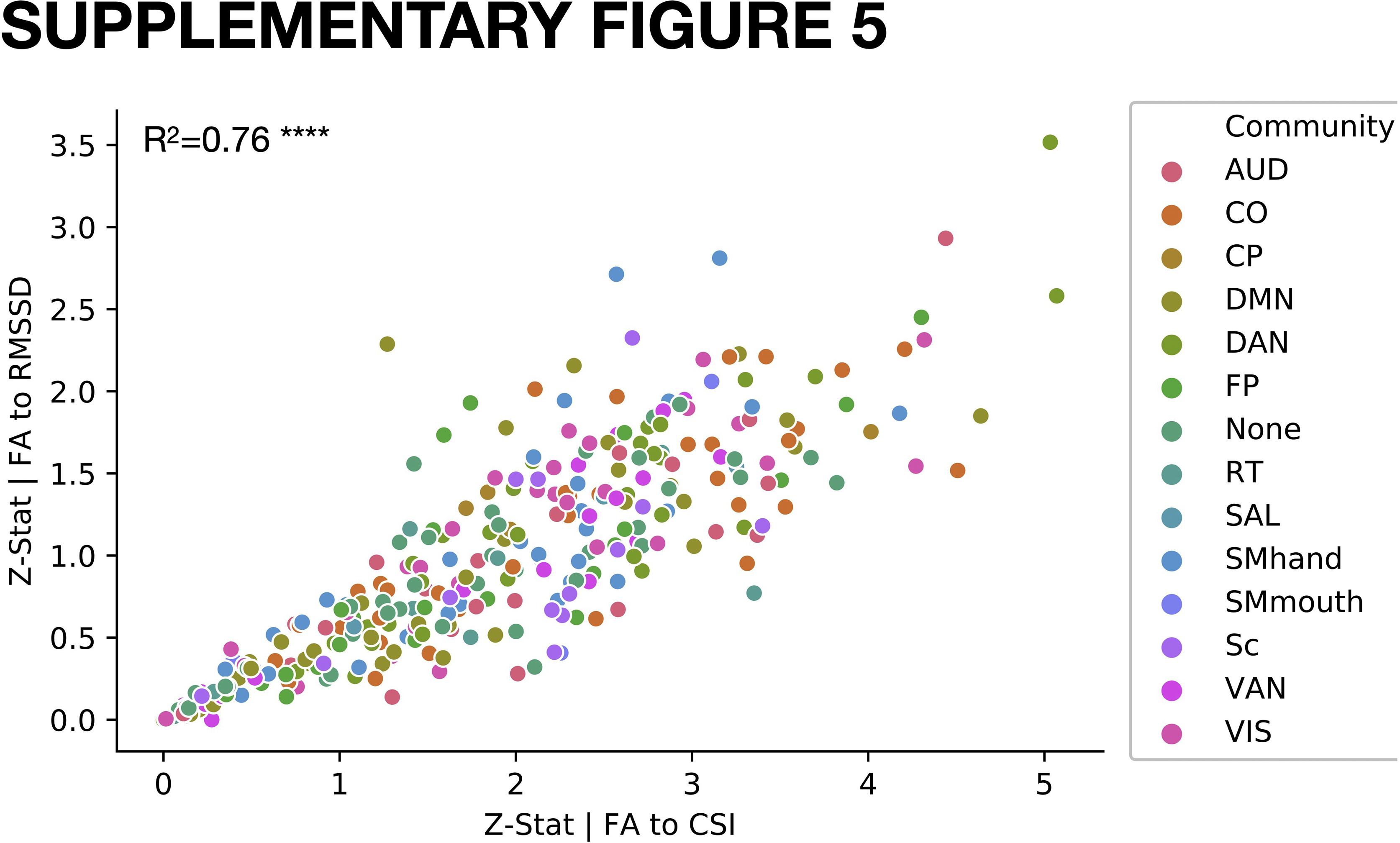

Supplement: Multimedia component 5 [file figs5.jpg]

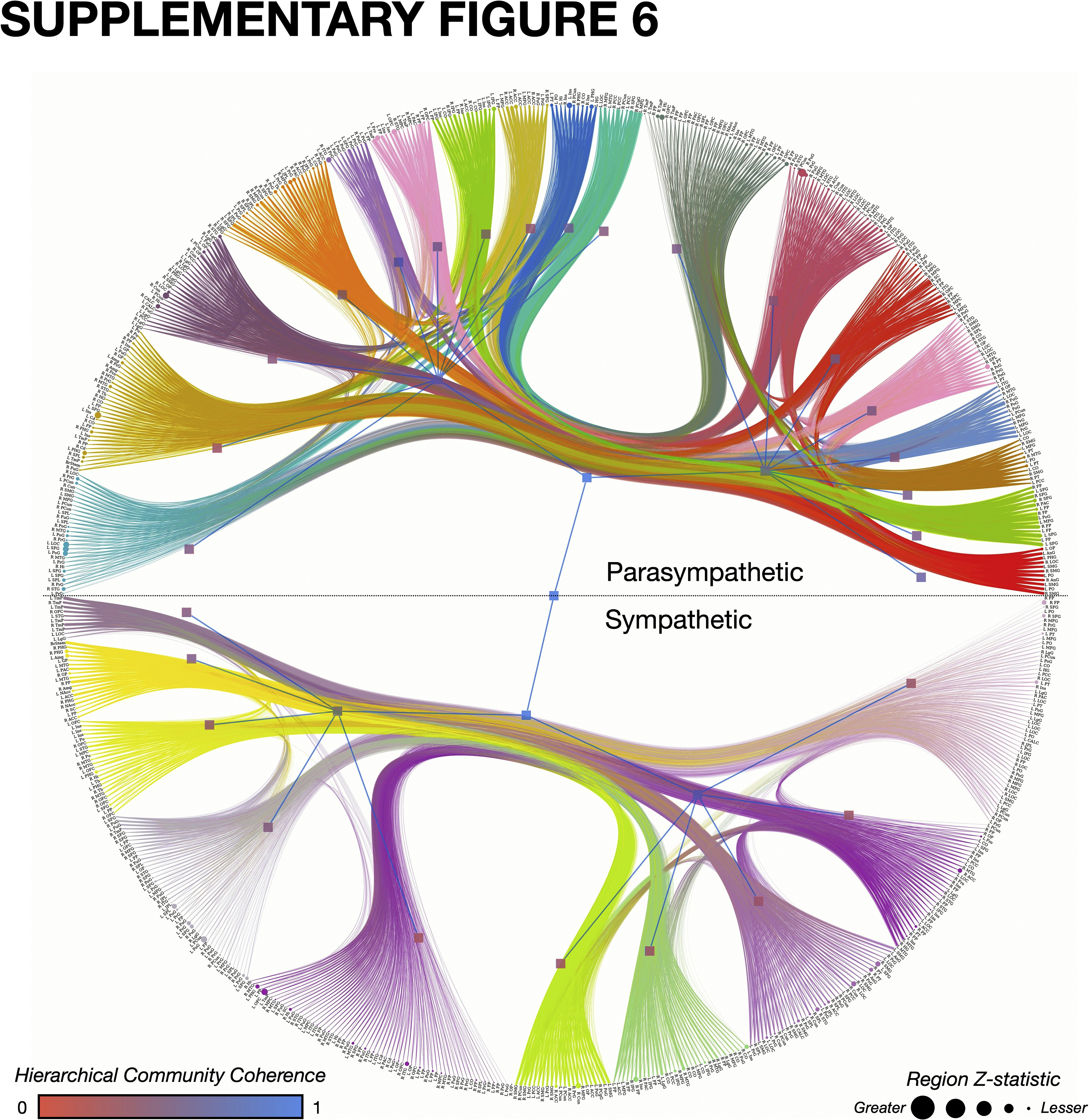

Supplement: Multimedia component 6 [file figs6.jpg]

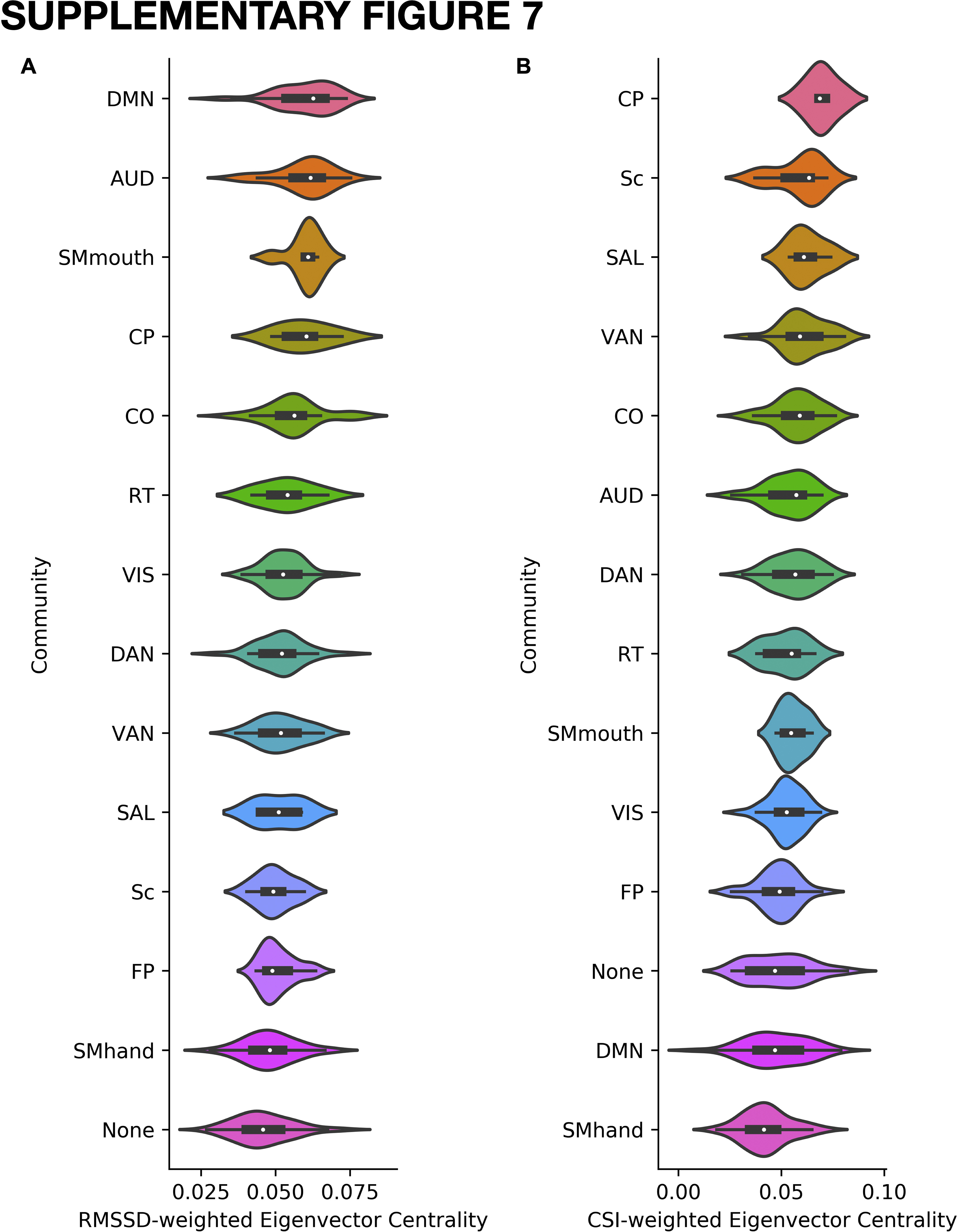

Supplement: Multimedia component 7 [file figs7.jpg]

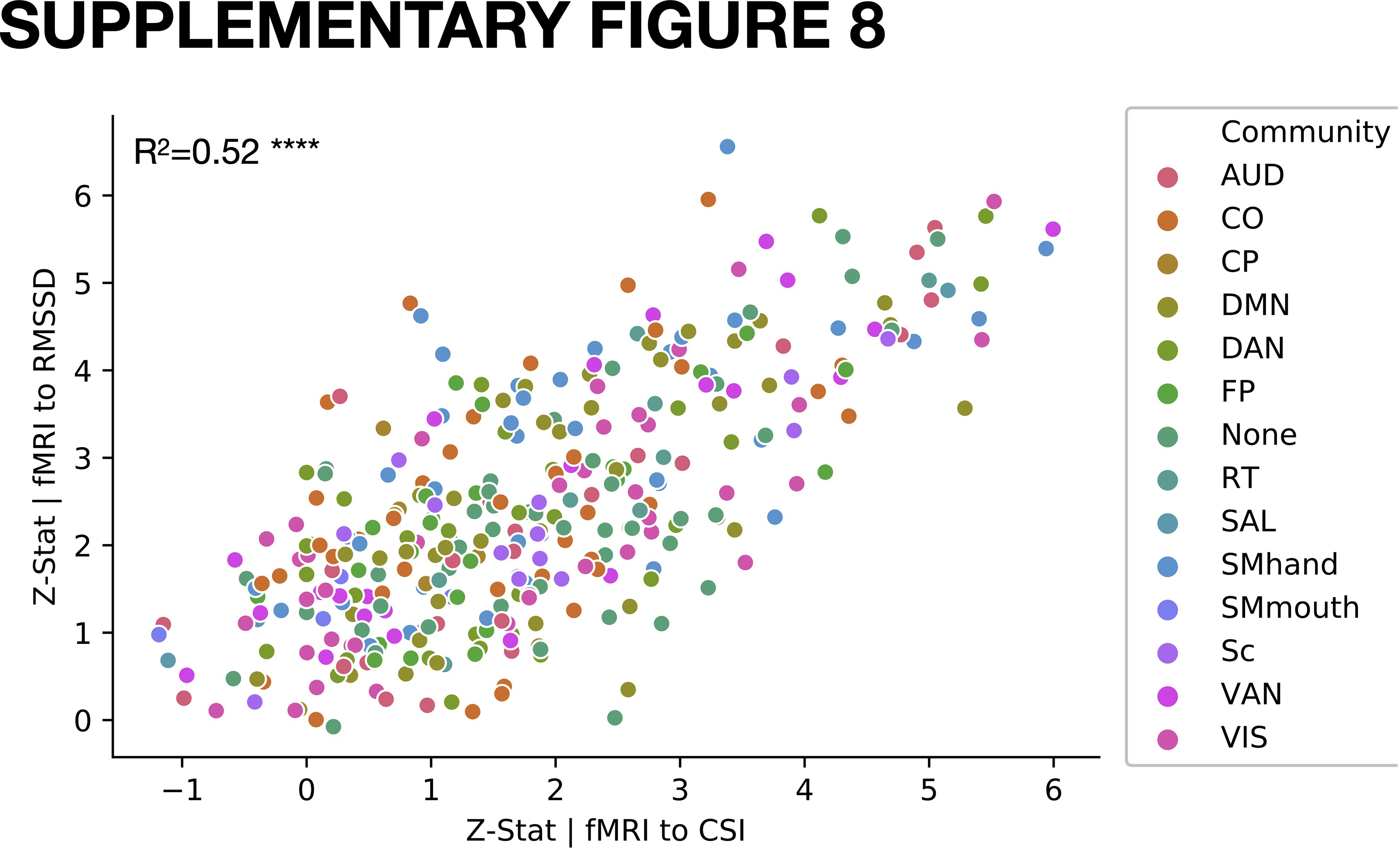

Supplement: Multimedia component 8 [file figs8.jpg]

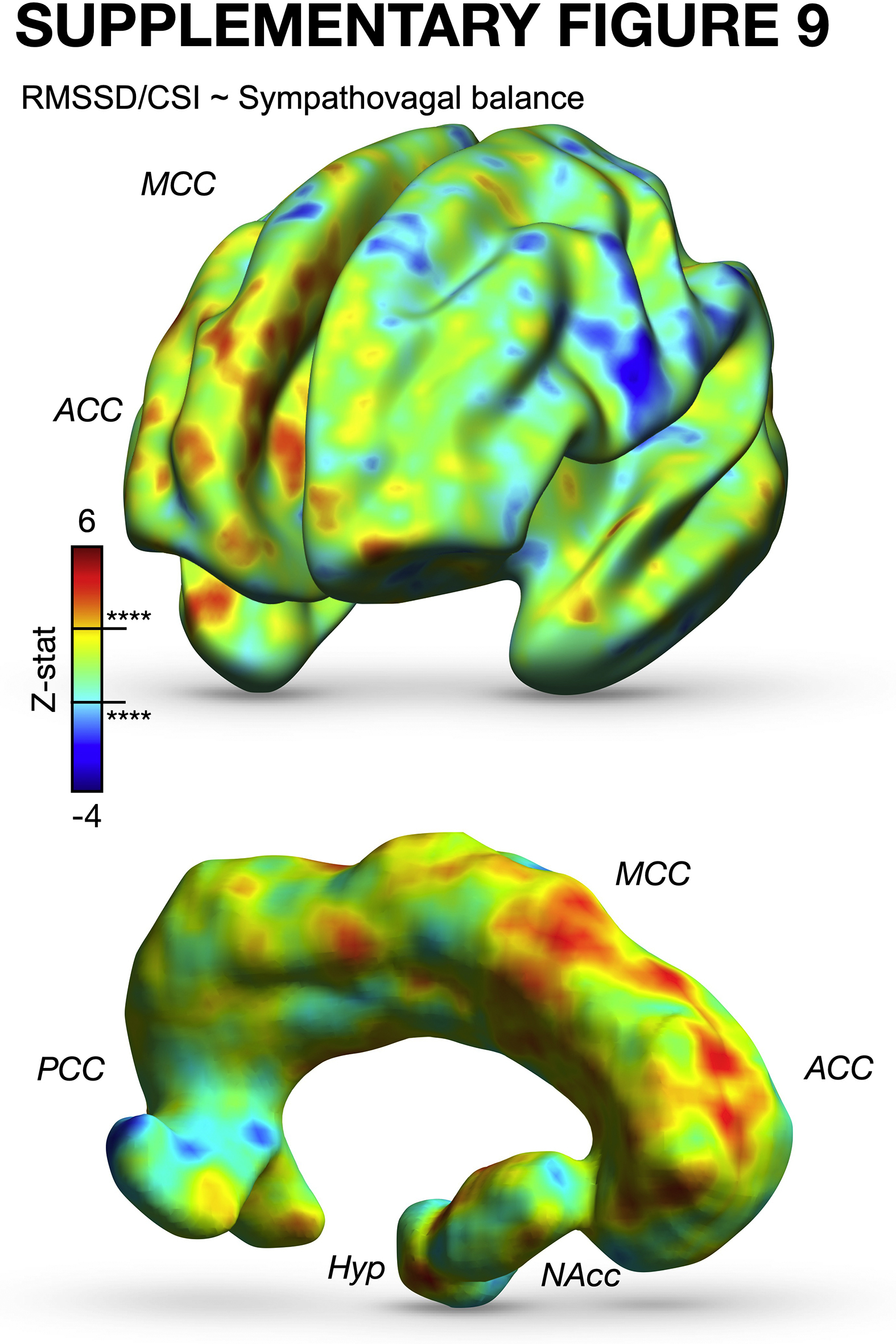

Supplement: Multimedia component 9 [file figs9.jpg]

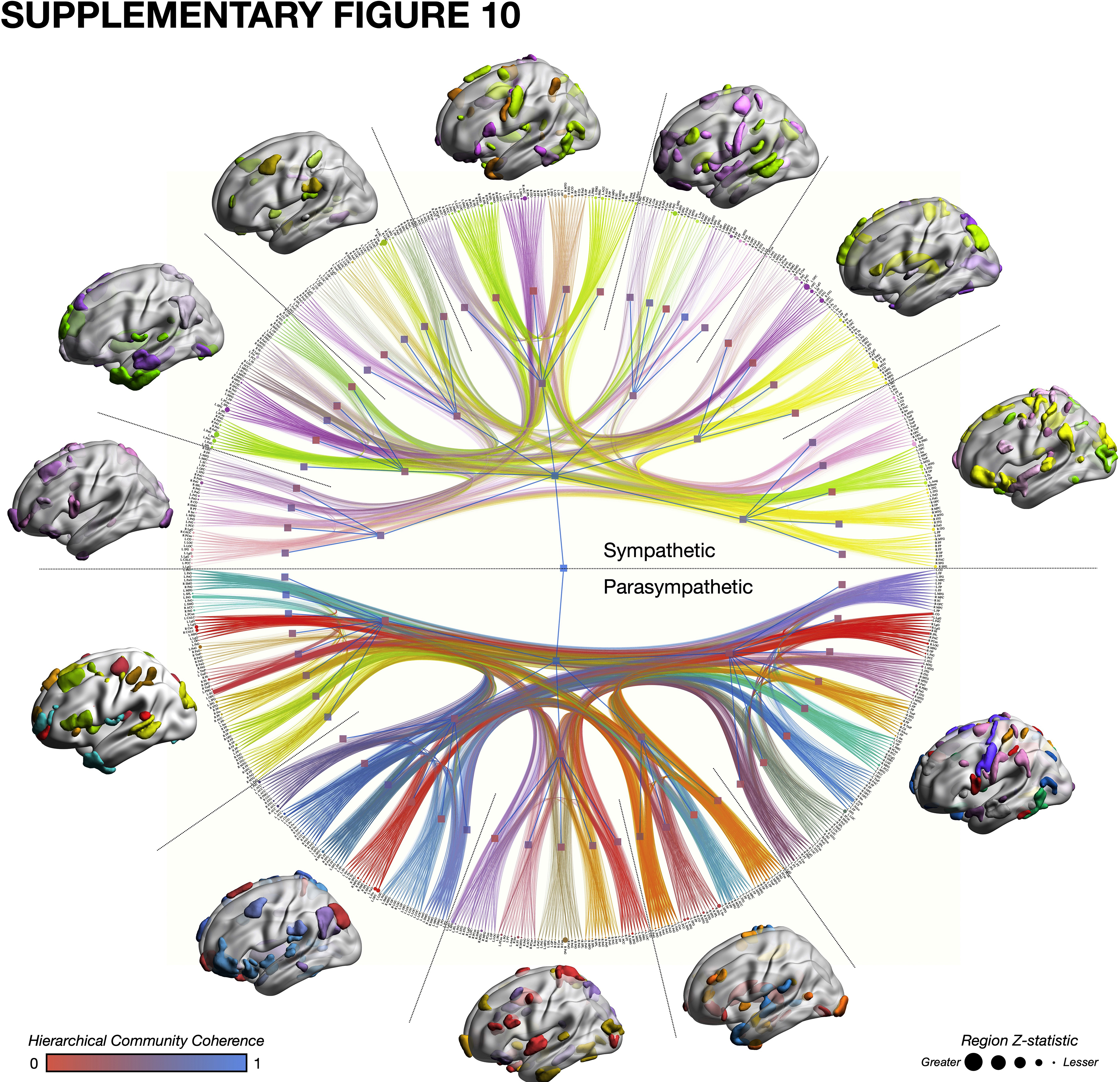

Supplement: Multimedia component 10 [file figs10.jpg]
